# Metabolic network reconstruction as a resource for analyzing *Salmonella* Typhimurium SL1344 growth in the mouse intestine

**DOI:** 10.1371/journal.pcbi.1012869

**Published:** 2025-03-11

**Authors:** Evangelia Vayena, Lea Fuchs, Homa Mohammadi Peyhani, Konrad Lagoda, Bidong Nguyen, Wolf-Dietrich Hardt, Vassily Hatzimanikatis

**Affiliations:** 1 Laboratory of Computational Systems Biotechnology, EPFL, Lausanne, Switzerland; 2 Institute of Microbiology, D-BIOL, ETH Zurich, Zurich, Switzerland; University of Delaware, UNITED STATES OF AMERICA

## Abstract

Nontyphoidal *Salmonella* strains (NTS) are among the most common foodborne enteropathogens and constitute a major cause of global morbidity and mortality, imposing a substantial burden on global health. The increasing antibiotic resistance of NTS bacteria has attracted a lot of research on understanding their modus operandi during infection. Growth in the gut lumen is a critical phase of the NTS infection. This might offer opportunities for intervention. However, the metabolic richness of the gut lumen environment and the inherent complexity and robustness of the metabolism of NTS bacteria call for modeling approaches to guide research efforts. In this study, we reconstructed a thermodynamically constrained and context-specific genome-scale metabolic model (GEM) for *S.* Typhimurium SL1344, a model strain well-studied in infection research. We combined sequence annotation, optimization methods and in *vitro* and *in vivo* experimental data. We used GEM to explore the nutritional requirements, the growth limiting metabolic genes, and the metabolic pathway usage of NTS bacteria in a rich environment simulating the murine gut. This work provides insight and hypotheses on the biochemical capabilities and requirements of SL1344 beyond the knowledge acquired through conventional sequence annotation and can inform future research aimed at better understanding NTS metabolism and identifying potential targets for infection prevention.

## Author summary

Nontyphoidal *Salmonella* strains are among the most ubiquitous enteropathogens that contribute significantly to global morbidity and mortality, posing a substantial burden on public health. While current antibiotic therapies have proven ineffective, identifying and targeting the metabolic processes essential for bacterial growth in the gut lumen may offer new intervention opportunities. The inherent complexity of the pathogen metabolism along with nutrient-rich and dynamic nature of the gut environment make the identification of new drug targets and therapeutic strategies cumbersome. In this work, we present a combined experimental and computational approach that incorporates *in vivo* and *in vitro* datasets and a high quality genome-scale metabolic model and advanced bioinformatics and optimization methods to help identify the metabolic capabilities and vulnerabilities of the pathogen in the gut of the mouse host. This interdisciplinary work can guide further studies towards a better understanding of the pathogen metabolism and, subsequently, the identification of targets for infection prevention.

## Introduction

Throughout the world, common pathogenic bacteria are becoming increasingly resistant to antibiotics, posing a significant threat to human health and well-being. This includes several types of pathogenic enterobacteria. *Salmonella enterica* is one of the most ubiquitous enteropathogens and is frequently associated with outbreaks of foodborne disease, causing 95 million cases and over 50,000 deaths [1] yearly worldwide. Non-typhoid *S. enterica* (NTS) infections like those caused by *S. enterica* subspecies 1 serovar Typhimurium range among the most common causes of diarrhea and inflict a substantial burden on global health [2]. So far, there is no vaccine to prevent NTS diarrhea, and antibiotic therapies have been proven ineffective [3]. Therefore, we need fresh approaches to improve therapy or to prevent the infection. A deeper understanding of the pathogen’s physiology could help to identify suitable targets.

After ingestion, NTS bacteria pass through the stomach and need to grow and survive in the lumen of the host’s intestine before they can invade the gut tissue and thereby cause diarrheal disease. However, in most cases, this initial gut-luminal growth is prevented by the dense intestinal microbiota via different mechanisms [4]. This phenomenon is commonly known as colonization resistance (CR). And while CR prevents *S.* Typhimurium growth in the intestinal lumen of most exposed hosts [5], in some cases, the pathogen can grow in the gut utilizing microbiota- [4,6] and host food-derived nutrients [6]. During this phase of initial growth in the gut lumen, the pathogen is thought to grow in an environment conditioned by the healthy gut microbiota and the healthy host [4]. Mouse infection experiments have identified metabolic enzymes which can contribute to initial growth. This includes the *hyb*-hydrogenase, the fumarate reductase (*frd*) which enable energy conservation by hydrogen/fumarate respiration and several transporters (i.e. *dcuA*, *dcuB*, *dcuC*) facilitating the export of succinate (which is the end-product of this form of anaerobic respiration) as well as the uptake of aspartate or malate, and the enzymes for malate and aspartate conversion into fumarate (*aspA*, *fumABC*) [6,7]. Genetic ablation of this metabolic pathway reduced the *S*. Typhimurium growth rate during initial growth by approximately 10%. Thus, additional nutrients and pathways must exist which can support gut-luminal growth of these mutants. It has been also shown that *S.* Typhimurium promotes its fitness by utilizing 1,2-propanediol, a microbiota-fermented product, through expression of the *pdu* operon [8], while mutants with no functional formate dehydrogenase (i.e., *ΔfdnGΔfdoG*) showed reduced fitness compared to the wildtype strain suggesting that the pathogen utilizes formate as an anaerobic electron donor [9]. Identifying the alternative nutrients and the metabolic pathways they fuel during the gut luminal colonization phase will help to devise ways to prevent *S.* Typhimurium diarrhea and possibly other enteropathogen infections that fuel gut luminal growth by similar mechanisms. However, the nutritional complexity of the gut environment, along with the inherent robustness of *S.* Typhimurium metabolism, require the employment of systems biology approaches to accelerate research efforts in this direction.

Genome-scale metabolic models (GEMs) are strain-specific databases of all known metabolic functions. The reconstruction of genome-scale models relies on the functional annotation of genes. GEMs have been widely used to study the metabolism of model organisms, such as *E. coli* [10] and yeast [11], and pathogens, such as *P. falciparum* [12], to identify metabolic host-pathogen interactions [13], drug targets [14], metabolic engineering strategies [15], and to predict microbiome composition [16], among others [17]. Genome-scale models, hence, represent a powerful framework to identify the essential biochemistry for non-typhoid *Salmonella* growth in the gut lumen which could be targeted to halt infections right during initial growth.

There are over 2,500 *Salmonella enterica* serovars that circulate globally. Out of these, *S. enterica* serovar Typhimurium, and in particular, the strain LT2 and the virulent strains SL1344 and ATCC14028, have previously served as tools to study the pathogen’s physiology and host-pathogen interactions. Most *Salmonella* studies on mouse models are based on the mouse-virulent strains 14028 or SL1344, with the latter being the topic of the present report. Although *S. enterica* strains share approximately 2,800 genes (i.e., the core genome) from a pan-genome of more than 10,000 genes [18,19], strains differ with respect to the accessory genes, with approximately 2,700 genes characterized as strain-specific [18]. Due to this diversity among *S. enterica* strains, to better simulate the complex host-microbiome-pathogen interactions and interpret the experimentally produced data, more rigorous modeling of the metabolism of SL1344 is needed. So far, modeling efforts have been mainly focused on describing the metabolism of the strain LT2 [20]. Recently, efforts were made to generate GEMs for 410 *Salmonella* strains, however, these models were curated to recapitulate some of the known metabolic features of *Salmonella* [21] rather than strain-specific traits.

Here, we introduce a context-specific and thermodynamically constrained genome-scale model, iNTS_SL1344 (Table 1), that captures the observed physiology of SL1344 and accounts for the metabolism of the strain in the gut of C57BL/6 mice associated with a low complexity microbiota (LCM) which had been established in earlier experimental studies [7,22]. We used a workflow to produce a high-quality GEM using bioinformatics, optimization methods and *in vivo* and *in vitro* experimental data. We started with the published GEM of the strain LT2, STM_v1_0 [20], as a base and we annotated the genome of SL1344 using the STM_v1_0 GEM and the KEGG [23–25] database. Single carbon and nitrogen source growth data under aerobic and anaerobic conditions were used to measure the catabolic capabilities of SL1344, while the extracellular space of the model was enriched with metabolites that are known to be associated with the physiology of the SL1344 in the murine gut [6,26–28]. We further amended the genome annotation of the model by suggesting catalyzing sequences [29] for 39 orphan reactions (i.e., reactions without genes associated with their functionality) in the genome of SL1344.

**Table 1:** STM_v1_0 and iNTS_SL1344 networks statistics.

	STM_v1_0	iNTS_SL1344
**Genes**	1,270	1,054
**Reactions**	2,545	2,735
Biotransformations	1,408	1,483
Transporters	792	872
Boundary	345	380
**Metabolites**	1,119	1,196
**Compartments**	Cytosol, Periplasm	Cytosol, Periplasm
**ΔG**^**o**^ **Metabolites**	77%	76%
**ΔG**^**o**^ **Reactions**	75%	68%

The model can be used as a scaffold to integrate experimental data [30,31] to study NTS metabolism in well-defined environments, i.e., i*n vitro* conditions, as well as in more complex and ill-defined environments, i.e., *in vivo* animal model systems, or to study host-pathogen and microbiota-pathogen interactions. In this study, we used the known metabolome of the murine gut, and we used the model to assess the nutritional requirements of SL1344 in a rich environment simulating the murine gut. We identified alternative *in silico* minimal media compositions that can support growth, ranked them with respect to biomass yield and experimentally verified some of our predictions. We identified the rate limiting metabolic enzymes for the growth of SL1344 and linked them to substrate availability. Finally, we use the model to analyze data from a recent transposon mutagenesis screen [6].

## Results

### Measuring the catabolic capabilities of *S.* Typhimurium SL1344 *in vitro*

The catabolic capabilities of *S.* Typhimurium SL1344 were analyzed *in vitro* using Biolog Phenotype MicroArrays (PMs) (S1 and S2 Figs) and in-house self-made microarrays (S3 and S4 Figs). The former allow convenient probing of *S.* Typhimurium SL1344 growth on a very large range of carbon or nitrogen sources. A drawback of PMs is that although the chemical nature of the respective compound (carbon or nitrogen) is revealed, the composition of the minimal media (substrates and concentrations) is not disclosed by the company as this is proprietary information. The in-house made assays are more labor-intensive but allowed us to probe growth at known substrate concentrations and complete control over the assay conditions.

**Fig 1:**
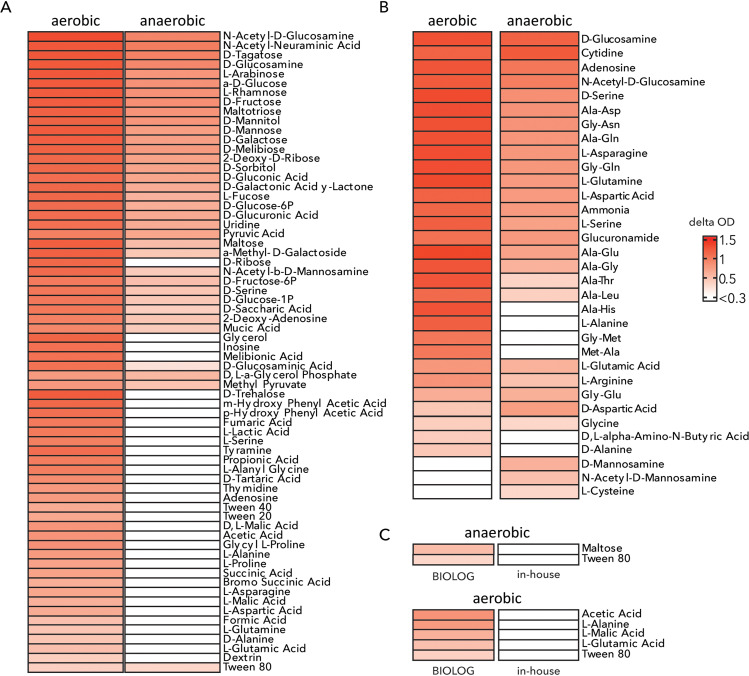
Catabolic capabilities of SL1344 *in vitro*. Compounds that serve as single (A) carbon sources and single (B) nitrogen sources under aerobic and anaerobic conditions, based on the Biolog Phenotypic MicroArrays PM1, PM2A and PM3B. (C) Discrepancies between the Biolog Phenotypic MicroArrays and the in-house made growth media. The complete datasets are shown in S1-S4 Figs.

The Biolog Phenotype MicroArrays PM1 and PM2A together contain 190 different carbon sources and PM3B 95 different nitrogen sources. Delta OD was calculated after 24 hours of growth. *Salmonella* SL1344 is a facultative anaerobe. Under aerobic conditions, more compounds can serve as carbon sources compared to the anaerobic conditions, as oxygen provides an excellent electron acceptor to generate ATP and for redox balancing. 68 compounds can serve as carbon sources under aerobic conditions and 35 compounds serve as carbon sources under anaerobic conditions (Fig 1A). We used the model to estimate to maximum ATP yield (mol ATP/mol C) in the presence and absence of oxygen for the carbon sources where growth was achieved only in aerobic conditions both *in silico* and *in vitro* (S1 Table). The model can achieve very low ATP yields on D-alanine, L-asparagine, and L-alanylglycine under anaerobic conditions. Increasing the allowable uptake flux for the carbon source or lowering the cell ATP requirements (i.e., no ATP for maintenance is required) is enough for the model to predict growth on these carbon sources anaerobically. However, growth is still infeasible when the rest of the compounds (i.e., glycerol, succinate, L-lactate and L-glutamate) serve as carbon sources (See S1 Text). This indicates that as oxygen is lacking under anaerobic conditions, alternative electron acceptors cannot support growth on the remaining carbon sources (S5 Fig). This was also verified by adding pseudo-reactions (e.g., NAD^+^ <-> NADH) to the model to allow for redox balance. After the addition of these pseudo-reactions, anaerobic growth could also be achieved on the remaining carbon sources.

Biolog PM3B was used to analyze the compounds serving as sole nitrogen source. Here, 30 compounds led to growth of SL1344 under aerobic conditions and 27 compounds served as sole nitrogen source under anaerobic conditions (Fig 1B). In the in-house made microarrays 41 different carbon sources were used of which 39 were also used in the Biolog assays. Here, five compounds which were carbon sources in the Biolog assay under aerobic conditions did not lead to the same result in the in-house made microarrays (Fig 1C). Under anaerobic conditions two compounds lead to growth in Biolog assays but not in the in-house made assays (Fig 1C). Such discrepancies between bacterial growth on Biolog versus in-house made media have been observed by other researchers before [32]. As concentrations and preparation of the media and the single carbon sources are known for the in-house made microarrays (but less well documented for the Biolog assays), we decided to use the data from the in-house made growth assays to further curate the model.

### A genome-scale metabolic model for *S.* Typhimurium SL1344

**Fig 2:**
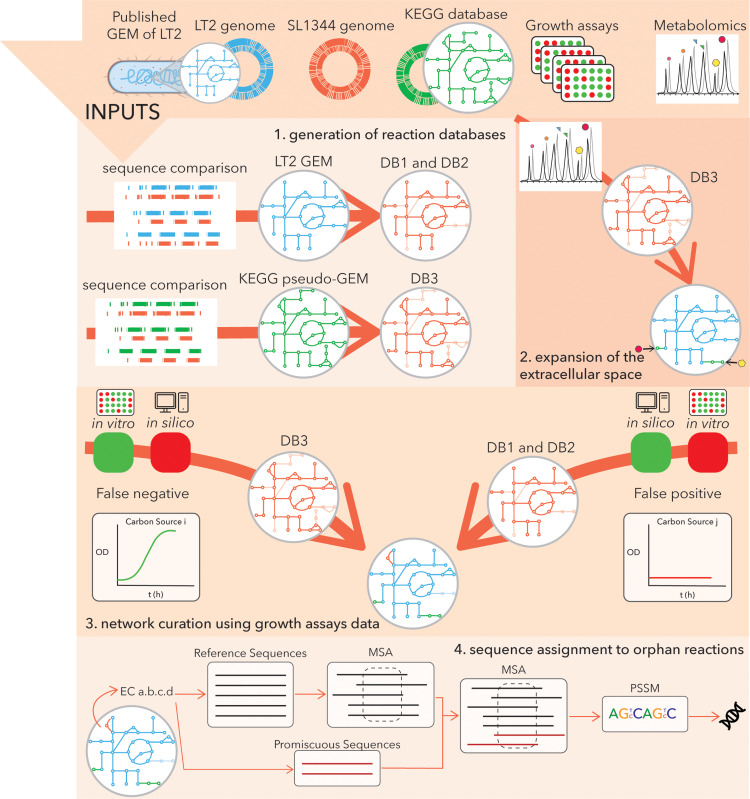
The workflow to generate the iNTS_SL1344 GEM. We reconstructed the iNTS_SL1344 GEM (2,735 reactions) starting from functional genome annotation with the KEGG database and the published GEM of the strain LT2, STM_v1_0 (2,545 reactions). This process outputted three reaction databases (DB1: 1,803 reactions, DB2: 364 reactions and DB3: 1,468 reactions) that were used for the network curation. We then used the STM_v1_0 as a basis and we characterized pathways to connect extracellular metabolites to the SL1344 metabolism. The expanded network was then compared against growth assays data and curated using the draft GEMs generated in the first step of the workflow and optimization methods. Finally, advanced bioinformatics methods were used to predict catalyzing genes for the newly added biochemistry.

To reconstruct the iNTS_SL1344 GEM we followed a workflow (Fig 2) that involves four steps and combines sequence annotation and optimization methods to exploit *in vivo* and *in vitro* data. The inputs to this workflow are the genome of SL1344 [33], the published GEM of the strain LT2 [20] and the genome [34] used to reconstruct it, metabolomics data from the murine gut, and *in vitro* growth assays data, as described in detail, below.

For the first step of the workflow, we generate three databases (S2 Text); the first (DB1 (1,803 reactions), S1 Data) and second (DB2 (364 reactions), S2 Data) databases are based on the STM_v1_0 GEM and contain the common biochemistry of the two strains and the reactions that are unique to LT2, respectively. The third database (DB3 (1,468 reactions), S3 Data) is based on the genome annotation of the SL1344 against the KEGG database, where we identify the genes in the genome of SL1344 with metabolic function. These three databases act as reaction pools for the curation of the STM_v1_0 to the iNTS_SL1344 network in the following steps.

For the second step of the workflow, we employ optimization methods to identify the minimal biochemistry required to consider extracellular metabolites that are relevant to the environment under study and model context-specific metabolism, i.e., the metabolic activity of *S. enterica* in the murine gut. To this end, the extracellular environment of the STM_v1_0 metabolic reconstruction was compared against a curated list of metabolites (S2 Table) that are suggested, based on *in vivo* metabolomics measurements and mutant screens [6,26–28], to be relevant to the physiology of non-typhoid *Salmonella* in the murine gut. For the work presented here, we have focused on 81 metabolites that have been implicated in the control of gut-luminal *Salmonella* growth by previous work and several additional metabolites that may be accessible to *S.* Typhimurium in the infected gut (S2 Table). This selection included well-established inorganic and trace elements and growth substrates like succinate, fumarate, malate, glucose, citrate and tatrate [35], as well as amino-acids that can be derived from food or the lysis of microbiota bacteria, L-lactate released from the infected gut and microbiota fermentation products released from the gut microbiota such as acetate, butyrate and propionate [36,37]. We also included metabolites which are known to be involved in the central carbon metabolism of *Salmonella* spp. including L-arabinose, D-fructose, D-glucose6-phosphate, D-mannitol, maltose, N-acetyl-D-glucosamine, glucoselysine and fructoselysine [38,39]. Finally, we included murein building blocks such as N-acetylmuramate, D-alanine which can be exchanged between gut-luminal bacteria [40], the immune-metabolite itaconate, which can be degraded by enzymes encoded in the *Salmonella rip*-operon [41] and bile salts that were found to promote gut-luminal growth of *S*. Typhimurium by direct- or indirect means [28].

It is worth noting that the concentration of these metabolites may vary significantly and is strongly influenced by diet, host and microbiome metabolism, as well as the feeding cycle. While the composition of mice food is standardized in terms of overall nutrient percentages, specific components (e.g., carbohydrate composition) can vary significantly, impacting nutrient availability in the gut lumen. Additionally, nutrient concentrations in the gut fluctuate in response to the host’s metabolism and feeding cycles. Similarly, the gut microbiome influences nutrient levels through its metabolic functions, with fluctuations driven by both nutrient availability and feeding cycles.

**Fig 3:**
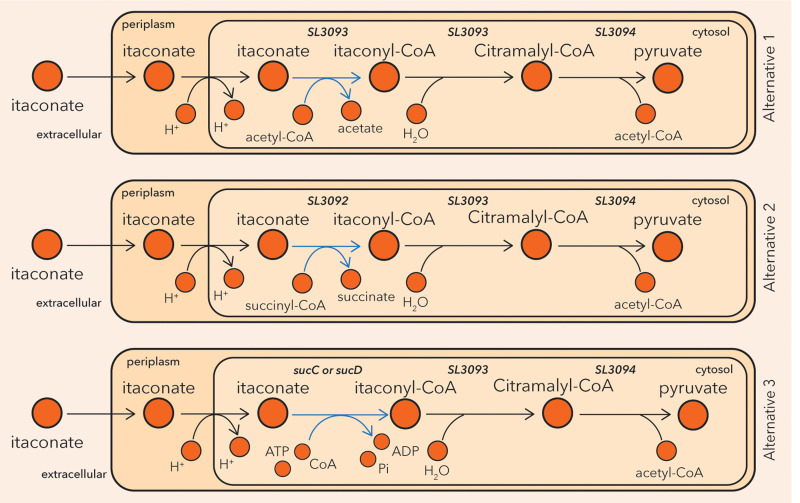
The three alternative pathways to connect itaconate to the metabolism of SL1344. All three pathways are included in the iNTS_SL1344 GEM, since catalyzing genes were annotated in the genome of SL1344 for all the constituent reactions. The three alternatives describing itaconate degradation to pyruvate differ in the first biotransformation (blue) where itaconate is converted into itaconyl-CoA. All three alternatives result in the same pyruvate yield (mmol pyruvate produced/ mmol itaconate).

Thirteen of these metabolites (S2 Table) were not part of the reconstruction. We thus characterized pathways (S3 Table) to account for the uptake and secretion of these compounds using the published algorithm redGEMX [42]. One of these metabolites is itaconate, which can be connected to the network using the DB3 as a database through a minimal set of three reactions. Our algorithm identifies three alternative solutions of three reactions that allow the degradation of itaconate by SL1344 that are combinations of five reactions (Fig 3). In all three solution sets itaconate is transformed to itaconyl-CoA, which then is hydrolyzed to citramalyl-CoA and finally breaks down to pyruvate and acetyl-CoA. The solutions differ in the first step of the conversion of itaconate to itaconyl-CoA. The mechanism is the same in the first and second alternatives; the CoA is transferred (Enzyme Commission number (EC) 2.8.3.-) to itaconate from acetyl-CoA and succinyl-CoA, respectively. The latter mechanism, where succinyl-CoA serves as the CoA donor, has been observed in *P. aeruginosa* and other bacteria [41,43]. In the third alternative, CoA is attached to itaconate through a ligase (EC 6.2.1.5). This mechanism is suggested for mammalian cells [41], however, the genes *sucC* and *sucD* have been annotated in the genome of SL1344 (SL1344_0721 and SL1344_0720) to catalyze the conversion of itaconate to itaconyl-CoA following this mechanism (Fig 3). The two last steps of the itaconate degradation pathway have been described for both bacterial and mammalian cells [41,43]. In this case, all the alternative pathways were added in the model, as the catalyzing genes for all the reactions involved were annotated in the SL1344 genome (see Materials and Methods).

**Fig 4:**
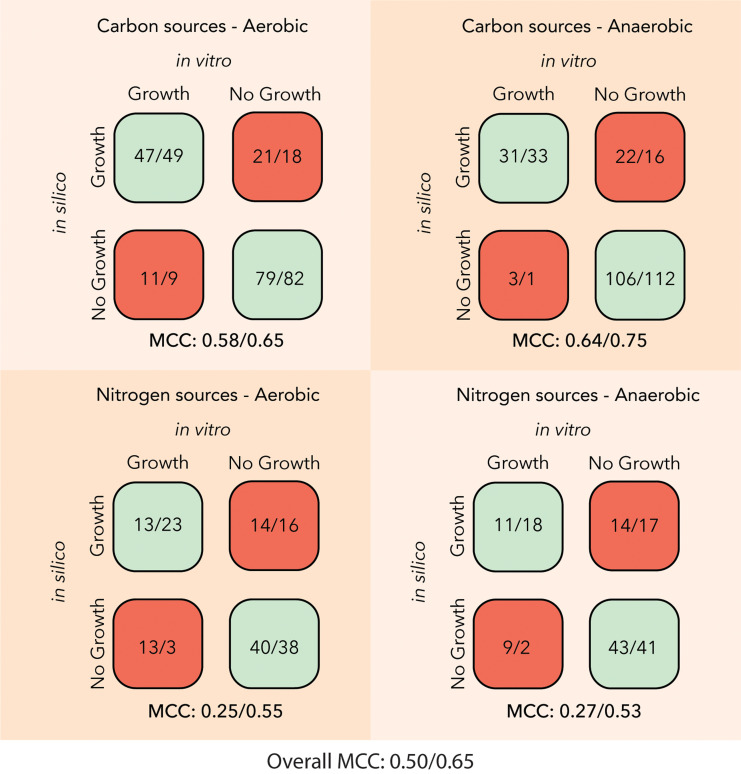
Comparison of the accuracy of growth predictions on different single carbon and single nitrogen sources for the STM_v1_0/iNTS_SL13344 GEMs. The iNTS_SL1344 has an improved average MCC score over the four datasets of 0.65 compared to 0.50 of the STM_v1_0 network and can thus capture more accurately the substrate utilization capabilities of the SL1344 strain.

For the third step of the workflow, the *in vitro* data from the Biolog Phenotype MicroArray Plates experiments along with the growth assays on the in-house made media were used to calibrate and curate the model (S6 Data). The SL1344 metabolism was simulated for the four different data sets, i.e., single carbon sources with ammonia as the nitrogen source and single nitrogen sources with pyruvate as the carbon source, under anaerobic and aerobic conditions. The model predictions were compared against the experimental observations, and the Matthews Correlation Coefficient (MCC) [44] was used as a metric of the accuracy of the model predictions (see Materials and Methods). The STM_v1_0 network scored an overall MCC of 0.50 across the four datasets (Fig 4). The network was most consistent with the carbon sources and anaerobic conditions dataset, whereas it shows greater disagreement with the nitrogen sources and aerobic conditions dataset.

We distinguished the two different types of incorrect model predictions. False Positives (FP) are cases, where the model predicts growth whereas the experiments suggest no growth. In case of the False Negatives (FN), i.e., the model predicts no growth whereas the experiments suggest growth. To reconcile the FP model predictions with the experimental data, reactions were removed from the model, whereas for the FNs biotransformation reactions were added parsimoniously to the model (S4 Table). After this curation, the iNTS_SL1344 shows an improved overall MCC of 0.65 across the four datasets (Fig 4), with the network scoring high accuracy with the carbon sources and anaerobic conditions dataset and considerably improved accuracy with respect to the nitrogen sources datasets.

For the remaining FN model inconsistencies (S6 Data), the insertion of reactions to curate the gap creates a FP model prediction. The remaining FP model inconsistencies can be split into different categories. For the first category of compounds, we could not identify any reaction to be removed from the network to curate those inconsistencies without creating FNs. The second category consists of compounds for which we could identify candidate reactions to be removed from the model, while genomic evidence suggests that they should be present. One example is 5-dehydro-D-gluconate. In the network, the compound is uptaken through a proton symporter (IndT) and is subsequently reduced to D-gluconate (IndO). In this case, a hypothesis we can make is that although the genes are part of the SL1344 genome, they are lowly expressed. Two interesting cases, cytosine and ethanolamine, were identified. The network falsely predicts growth when either of the two compounds is used as a nitrogen source. The inconsistency can be resolved by eliminating *in silico codA* and *eutBC*, respectively, from the model. The genome annotation suggests these genes are part of the SL1344 genome, and thus the genes were not removed. However, it has been observed that the presence of cytosine decreases the activity of the catalyzing enzymes cytosine deaminase [45] and ethanolamine ammonia-lyase is coenzyme B12-dependent [46]. Thus, the inconsistency between the experiments and the model predictions may be due to regulation or the media composition.

Lastly, we used the computational annotation tool BridgIT^+^ [29] to further curate the genome annotation of iNTS_SL1344. The tool identified candidate catalyzing genes for 39 orphan reactions (S5 and S6 Tables). The orphan reactions can be products of previous gap-filling efforts of the STM_v1_0 network and are thus present in the network to allow the simulation of an observed phenotype. For example, we identified the orphan reactions MDRPD, ALATA_L, GCALDD, MTRK, PMDPHT and DNMPPA (S7 Table) as essential for growth on glucose minimal media and aerobic conditions. Alternatively, an orphan reaction can have been experimentally observed while the catalyzing sequence has remained unknown. Finally, orphan reactions were added to the network to account for the uptake and secretion of thirteen extracellular metabolites and to reconcile the model predictions with the Biolog data. In total, we suggested 37 genes for these orphan reactions (S5 and S6 Tables). For the added reactions that were part of DB3, we used the gene rules as described in DB3 (S8 Table).

### *In silico* identification of the nutritional requirements for NTS growth in the murine gut

**Fig 5:**
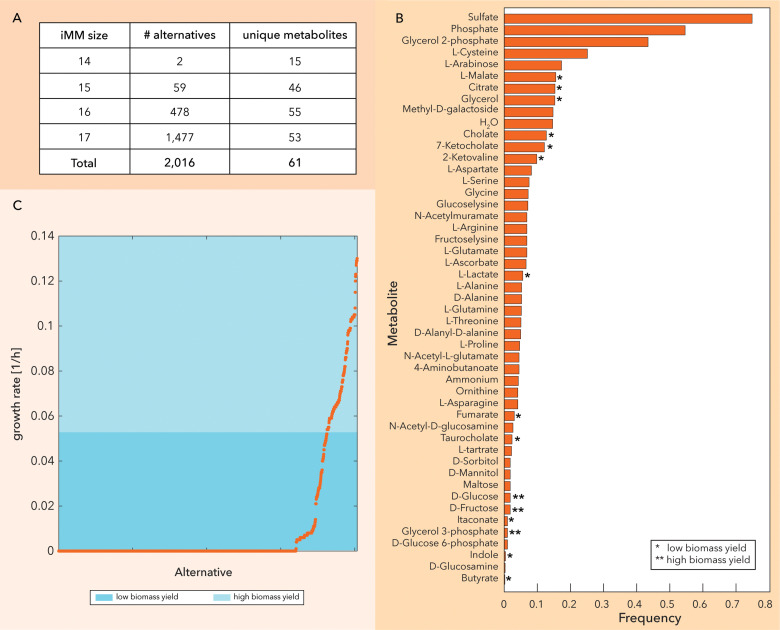
*In silico* minimal media analysis. (A) Statistics for the generated iMMs. The algorithm identified 2,016 minimal media compositions of the minimal (14 compounds) and the three subsequent sizes among 61 metabolites. (B) and (C) The iMMs were ranked according to the biomass yield. Certain metabolites appear only in high or only in low and yield iMMs.

We used the curated and context-specific metabolic network to identify metabolites essential for the growth of SL1344 in a rich environment simulating the murine gut. An *in silico* Minimal Media (iMM) analysis [12,42,47] was performed for anaerobic conditions to suggest putative nutrients for SL1344 within a list of 81 metabolites relevant to NTS physiology in the murine gut environment. This relevance was inferred from previous publications measuring fitness defects of *S*. Typhimurium mutants [6,9,48,49] and from experiments assessing gut-luminal substrates which may support *S*. Typhimurium growth[6,26–28]. Starting from the rich environment, including inorganics, amino acids, carboxylic acids, sugars, and bile acids, we generated all alternative iMMs of minimal size, i.e., sets of the minimal number of compounds required to achieve certain growth, that was 14 metabolites in this case, and the three subsequent sizes (i.e., 15, 16 and 17 media components) (Fig 5A and S8 Data). We identified 2,016 iMMs in total that involved 61 metabolites. L-histidine is part of all alternatives since SL1344 is a histidine auxotroph. In the high-frequency metabolites (i.e., metabolites that appear frequently in the different alternatives) (Fig 5B), sulfate and L-cysteine can act as the sulfur source in the different alternatives. Our model also shows that in principle, phosphate anions can be replaced by organic phosphate sources such as glucose 2-phosphate and less frequently, glucose 3-phosphate or glycerol 3-phosphate (Fig 5B), if such compounds are available. It has been recently shown that mixed-acid fermentation of hexoses, such as glucose and fructose, fuels the initial growth of SL1344 in the murine gut [50]. Furthermore, SL1344 has been shown to utilize D-glucose and D-fructose *in vivo* within diverse microbiota contexts [51], underlining the importance of these compounds. Finally, *Salmonella* switches to gluconeogenesis in the inflamed gut. [50] Notably, glycerol-3-phosphate can fuel both glycolysis and gluconeogenesis, further supporting its relevance to study.

Then, for each of the iMMs, we constrained the upper limit of the uptakes in the model to equimolar total carbon (i.e., 60 mmol C/gDW-h) and equimolar total nitrogen (i.e., 2 mmol N/gDW-h) uptake and simulated the optimal growth. This allowed us to rank the different iMMs based on the biomass yield (Fig 5C) and identify high and low-yield substrates. Under these conditions, the simulated growth rate is directly proportional to the biomass yield, as the model assumes optimal utilization of the available resources. Consequently, growth rate serves as an effective proxy for the final biomass produced, facilitating the distinction between high- and low-yield substrates. The iMMs that ranked higher contain glucose, fructose and glycerol-3-phosphate as carbon source. On the other hand, butyrate, itaconate, indole, L-lactate, citrate, L-malate, fumarate, 2-ketovaline, glycerol, and bile acids only appear in iMMs that are classified as low-yield media. We also identified the sets of minimal secretions for each of the iMMs at the optimal biomass yield, which is here defined as grams of dry weight per mmol of carbon uptake (S8 Data). The number of minimal secretions (organic and inorganic compounds) varies from five to fourteen metabolic byproducts at the optimal yield. Overall, we identified 79 putative metabolic byproducts, 42 of which appear only with low-yield media (S7 Fig). Among the predicted byproducts, succinate, formate, ethanol, acetate and lactate have been identified as byproducts of SL1344 metabolism in LCM mice in a recent study [50]. In our model, lactate is predicted as a putative byproduct only in low-yield media where methyl-D-galactoside, glutamine and glycine are the carbon and nitrogen sources, while the other compounds frequently appear among the predicted byproducts. To provide further insights, we pinpointed the substrate – byproduct associations for these 5 compounds (S8 Fig).

**Fig 6:**
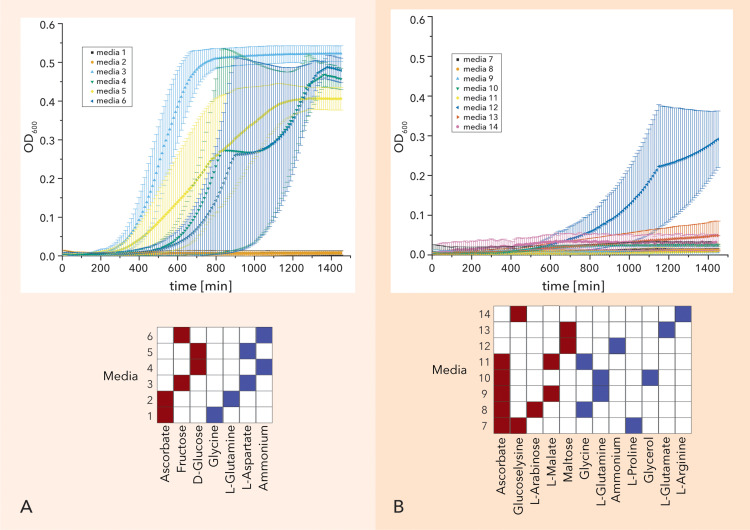
Experimental validation of predicted iMMs. (A) Predicted high yield minimal media and (B) Predicted low yield minimal media and the media composition (in red the assumed carbon source and in blue the assumed nitrogen source.

To validate the iMM predictions, we performed a series of experiments. More specifically, we tested *in vitro* iMMs from each yield regime. Based on the optical density measurements, the model can correctly differentiate the high-yield substrates from the low yield, with the exception on the predicted high-yield iMMs containing ascorbate (based on the experiments performed ascorbate cannot serve as single carbon source) (Fig 6). Notably, medium #12 (Maltose and Ammonium) yielded a higher optical density compared to other tested media in the low-yield regime. This can be attributed to the specific combination of carbon and nitrogen sources. In contrast, medium #13 (Maltose and Glutamine) resulted in much lower growth, underscoring the significant impact of nitrogen source growth.

### *In silico* gene knockout studies identify key metabolic genes for the SL1344 growth

**Fig 7:**
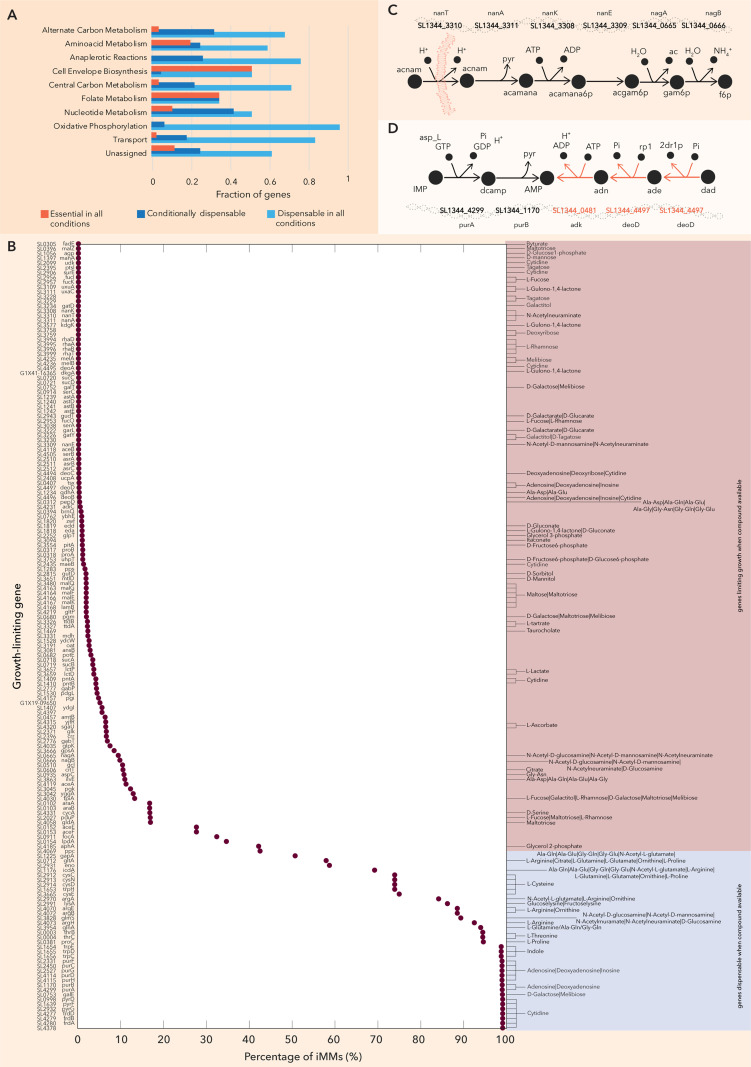
*In silico* gene essentiality analysis. (A) Genes are classified as essential in all conditions, conditionally essential and dispensable in all conditions. The distribution of the genes in these three categories in each metabolic subsystem hints at the pathway utilization under the conditions under study and the robustness of the subsystem. (B) Percentage of iMMs (x-axis) in which a mutant (y-axis) has decreased fitness. This analysis allows to map gene essentiality to substrate availability. (C) The genes SL1344_3308, SL1344_3310, and SL1344_3311 are responsible for the uptake of N-acetylneuraminate (acnam) and its conversion to N-acetyl-D-mannosamine (acamana) and finally of N-acetyl-D-mannosamine (acamana6p), whereas the genes SL1344_3309, SL1344_0665 and SL1344_0666 catalyze the further conversion of N-acetyl-D-mannosamine 6-phosphate to D-Glucosamine 6-phosphate (gam6p) and finally to D-fructose 6-phosphate (f6p) and are always essential when either N-acetylneuraminate or N-acetyl-D-mannosamine are in the media. (D) The genes SL1344_4299 and SL1344_1170 that catalyze the conversion of IMP to AMP with adenylosuccinate (dcamp) as intermediate become dispensable when adenosine (and) or deoxyadenosine (dad) are in the media, since AMP can be synthesized through nucleotide salvage pathway reactions (orange). All simulations are performed for anaerobic conditions.

We used the model to explore *in silico* the growth supporting metabolic genes of *Salmonella* in the murine gut environment and gain mechanistic understanding of how the deletion of a gene affects growth. The curated iNTS_SL1344 model was used to predict the growth defect of metabolic gene knockouts (KO) on the collection of all the generated iMMs and the minimal media used for the single carbon and nitrogen source growth assays and anaerobic conditions (only the TPs), i.e., in 2,082 different media conditions in total. We then assigned genes in different categories depending on the metabolic subsystems they belong to identify the essential metabolism for NTS growth. Analysis of the results allows us to identify genes dispensable in all the conditions examined in this study, i.e., genes whose individual deletion reduces growth less than 10% of the optimal WT growth, essential genes whose individual deletion reduces growth more than 90% of the optimal WT growth in all the conditions examined in this study, and genes that conditionally affect growth. More specifically, *in silico* 763 of the 1,054 genes in the iNTS_SL1344 network are dispensable in all conditions. The number of dispensable metabolic genes is comparable to previously reported Figs for known metabolically robust bacteria such as *E. coli* (1,279 out of the 1,503 genes in the iML1515 GEM are dispensable for growth on 15 different single carbon source minimal media [10]) and *B. subtilis* (675 metabolic genes in the iYO844 GEM are dispensable for growth on rich media [52]). More than 90% of the genes assigned to the oxidative phosphorylation subsystem fall into the category of dispensable genes (Figs 7A and S9). In all main metabolic subsystems, apart from the cell envelope biosynthesis, more than half of the assigned genes are dispensable in all conditions. This fact highlights the metabolic robustness of NTS bacteria. In some cases, the deletion of the gene does not affect the growth thanks to the presence of isoenzymes. Such an example is the biotransformation of 3-Phospho-D-glycerate to D-Glycerate 2-phosphate, that is essential under glucose minimal media conditions. This biotransformation can be catalyzed by SL1344_3670 or SL1344_4512 or SL1344_0749 (*gpmA or gpmB or pmgI)*, thus in a single gene deletion analysis none of the three possible catalyzing genes appear as essential. However, this is not necessarily physiologically relevant, since in reality not all gene products that can perform the same biotransformation are equally efficient and they might be expressed under different environmental conditions.

The single deletion of a set of 126 metabolic genes highly impacts growth in all conditions and can be considered as the minimal metabolic genome that needs to be functional for growth in any growth condition. Half of the genes catalyzing the cell envelope biosynthesis belong in this category (Fig 7A and S9 Data) since the cells need to synthesize *de novo* the complex lipopolysaccharides of the cell membrane from simpler molecules.

Finally, the remaining 165 genes included in the iNTS_SL1344 whose absence reduces growth in some media compositions only. This set of genes is referred to as *conditionally dispensable.* The set of conditionally dispensable genes allows us to map substrate-specific essential pathways [53] (Fig 7B). For example, the genes SL1344_3308 (*nanK*), SL1344_3310 (*nanT*), and SL1344_3311 (*nanA*) are always essential in the model when N-acetylneuraminate is in the media (Fig 7B), indicating that there is no alternative pathway to catabolize this compound. The genes catalyze the transport of N-acetylneuraminate and its conversion to N-acetyl-D-mannosamine 6-phosphate (Fig 7C). Similarly, the genes SL1344_3309 (*nanE*), SL1344_0665 (*nagA*), and SL1344_0666 (*nagB*) catalyze the further conversion of N-acetyl-D-mannosamine 6-phosphate to D-fructose 6-phosphate (Fig 7C) and are always essential when either N-acetylneuraminate or N-acetyl-D-mannosamine are in the media (Fig 7B). Reduced fitness of *Δnan* or *Δnag* mutants can hint utilization of the substrates. On the other hand, genes in the nucleotide biosynthesis pathways, such as SL1344_4299 (*purA*) that catalyze the production of adenylosuccinate, an intermediate in the synthesis of AMP from IMP, become dispensable when adenosine or its precursor deoxyadenosine is present in the media (Fig 7D). Thus, unimpacted *Δpur* mutant fitness could suggest purine availability in the environment.

We compared the data from our computational growth analysis to the data from a recent transposon mutagenesis experiment, which studied the fitness of over 700 *S*. Typhimurium SL1344 mutants during the initial growth (.i.e., days 1 and 2 post infection) in the gut of gnotobiotic mice [6]. This allows us to identify substrate utilization in the murine gut but also the alternative metabolic pathways or isoenzymes that enable the survival of the mutants. We focus on mutants in the screen with significantly (p<0.05, Mann-Whitney U test) impaired or enhanced initial growth (S9 Table).

More specifically, the *Δfrd* mutant is strongly attenuated *in vivo* (i.e., competitive index (CI) ≈ 0.03 at 1 day post infection). *In silico,* the *Δfrd* mutants cannot survive in any of the minimal media tested, with the exception of minimal media containing cytidine (Fig 7B). Alternatively, in the rich extracellular environment (S1 Table), uracil can be utilized by the mutant. Further computational analysis reveals that the *frd* mutation affects the biosynthesis of the biomass building blocks UTP, CTP, dCTP, dTTP, and UDPglucose. *In vivo*, deletion of *sucCD* (CI ≈ 0.71) or *mdh* (CI ≈ 0.79) has a minor effect on the mutant fitness. Deletion of these genes is growth-reducing *in silico* if citrate and fumarate or malate, respectively, are the main carbon sources, but the genes are redundant when sugars and amino acids are available (Fig 7B). Thus, for these mutants we observed a good fit between the *in vivo* data and the *in silico* results.

While *ΔaspA* mutants show an intermediate level of attenuation *in vivo* (CI ≈ 0.15), the gene is dispensable in all minimal media compositions tested *in silico.* We further explored this discrepancy. The gene product of *aspA* is responsible for the conversion of aspartate to fumarate, which can act as an electron acceptor *in vivo* [6]. *In silico*, the *ΔaspA* mutant converts aspartate to fumarate in three steps; aspartate is converted to oxaloacetate (*aspC*), then malate (*mdh*) and finally to fumarate (*fumA or fum or fumC*). Alternatively, malate (as a precursor for fumarate) can be derived from pyruvate (*sfcA*). The *ΔfumΔaspA* mutant has a decreased fitness by 20% compared to the wildtype *in silico*. The *in silico* deletion of *fumA*, *fumB* or *fumC* genes does not have any effect on any of the growth rate (the gene-reaction rule is *fumA or fumB or fumC*). This is line with the mutant screen data since the *fumAC* (CI ≈ 0.91) and *fumB* (CI ≈ 0.67) mutants have only a minor growth defect. We conclude that *in vivo*, the redundant fumarate synthesis pathways offered by *aspC*/*mdh*/*fum* or by *sfcA* may not be fully able to replace *aspA*. This could explain the apparent discrepancy between the *in vivo* and the *in silico* results and could be tested experimentally in future work.

Similar considerations apply to the *dcu* transporters, which are required to secrete succinate and import aspartate or malate. *In vivo* a triple mutant lacking *dcuA*, *dcuB* and *dcuC* was strongly attenuated (CI ≈ 0.01 to 0.001) [6], while the *dcuB* (CI ≈ 0.53) mutant shows mild attenuation. *In silico* the genes are dispensable in all the minimal conditions studied since the gene-reaction rule is *dcuA or dcuB or dcuC or SL1344_3564*. Finally, the *ΔglnA* mutant has increased fitness suggesting that L-glutamine or dipeptides containing it are present in the environment (Fig 7B). Overall, we conclude that our curated GEM is consistent with the growth defects of *S*. Typhimurium mutants in the gut of a gnotobiotic mouse model.

## Discussion

In this work, we reconstructed a context-specific and thermodynamically curated GEM for *S.* Typhimurium SL1344 using advanced bioinformatics and optimization methods and experimental data. The iNTS_SL1344 network shows an improved performance with respect to single carbon and single nitrogen source utilization compared to the previously published GEM of the strain LT2 [20]. The overall MCC of iNTS_SL1344 across the examined datasets is equal to 0.65 compared to 0.50, whereas the MCC for single carbon source utilization under anaerobic conditions is 0.75 compared to 0.64. Higher MCC (i.e., MCC closer to 1) is not achieved for different reasons. Some of the metabolites included in the dataset are included in gene-enzyme-reaction databases but no reactions are associated with them (e.g., Tween 20), thus we cannot characterize any pathway for their catabolism using reported biochemistry. FP model predictions can arise due to the lack of transporters in the organism, (the metabolite is not transported in the cell but the biochemistry to catabolize it exists). For example, no specific transporter was annotated [54] for 2-aminoethanol, dihydroxyacetone, itaconate, L-tartaric acid and N-acetyl-L-glutamic acid, compounds for which our model incorrectly predicts growth. Furthermore, GEMs do not consider regulation or toxicity, which can prevent growth on a substrate and thus result in FP. Finally, as shown here and in other studies [32] discrepancies can occur between house-made media and Biolog plates.

We used the model to study the nutritional requirements of SL1344 in a rich environment simulating the murine gut lumen, and we identified high and low-yield substrates. Results of this study were experimentally tested, with the directionality of the model prediction matching the *in vitro* results. Lastly, we studied the growth limiting metabolic genes of SL1344, and we mapped them to substrate availability. The results of this analysis are subject to the extracellular metabolites considered, i.e., inclusion of more extracellular metabolites can result in different minimal media compositions that fuel different metabolic pathways and thus reveal more metabolite-dependent growth limiting genes.

We demonstrated that the gene essentiality results can be employed to interpret results from *in vivo* mutant fitness screens (like transposon mutagenesis screens identifying genes promoting growth at particular sites within the infected host [6,55]). We speculate that integrating data from our curated GEM, transposon mutagenesis, mutant fitness experiments and analysis of metabolite concentrations at the site of interest might allow us to decipher the pathogen’s growth physiology at a given site within the host and to suggest the composition of the complex media which could be used to simulate this physiology *ex vivo*. These results can also shed light on alternative pathways to perform the same metabolic task and thus reveal new targets for combined inhibition (i.e., higher order gene KOs) which could be used to prevent pathogen growth and allow new therapeutic strategies against NTS infections.

The model and the analysis framework presented here, available through Zenodo [56], are valuable resources that can facilitate efforts to obtain a better understanding of NTS metabolism under different conditions, i.e., in the human gut lumen. The pipeline followed here can be applied to study the metabolism of any organism with a sequenced genome.

## Materials and methods

### Ethics statement

All *in vivo* data were derived from published work. No new *in vivo* data were performed for this work.

### Thermodynamic curation of the genome-scale model

The thermodynamic curation [57,58] of the iNTS_SL1344 GEM aims to include thermodynamic information, i.e., the Gibbs free energy of formation for the compounds and the corresponding error for the estimation, into the model. We used the pipeline to estimate this information as described before [42]. Briefly, The .${\text{Δ}}_{\text{f}}{\text{G}}^{0\text{'}}$. of metabolites was estimated with the Group Contribution Method [58]. Subsequently the estimation of ${\text{Δ}}_{\text{f}}{\text{G}}^{0}$of metabolites and ${\text{Δ}}_{\text{r}}{\text{G}}^{0}$of reactions and the simulations with thermodynamic constraints were performed with the matTFA toolbox [59]. The thermodynamic properties of the compartments of *S.* Typhimurium cells have been integrated with the model (S10 Table).

### Measuring the catabolic capabilities of SL1344

The Biolog Phenotype MicroArrays (PMs) PM1, PM2A and PM3B were used to determine catabolic capabilities of *S*. Typhimurium SL1344 M678, an SL1344 strain which is histidine prototroph, under aerobic and anaerobic conditions (gas atmosphere 7% H_2_, 10% CO_2_, 83% N_2_). Catabolic capabilities were determined measuring tetrazolium dye absorption at 590 nm of Biolog redox dye mix A using a tecan plate reader Infinite M200 Pro.

Bacteria were grown over night in minimal media M9 (47.75 mM Na_2_HPO_4_ ∙ 7H_2_O, 22.04 mM KH_2_PO^4^, 8.55 mM NaCl, 100 µM CaCl_2_, 2 mM MgSO_4_) supplemented with Wolin’s trace elements (13.4 mM EDTA, 3.1 mM FeCl_3_-6H_2_O, 0.62 mM ZnCl_2_,76 nM CuCl_2_-2H_2_O, 42 nM CoCl_2_-2H2O, 162 nM H_3_BO_3_, 8.1 nM MnCl_2_-4H_2_O) and 312.42 µM sodium fumarate, which serves as an electron acceptor under anaerobic conditions [6] with 22mM glucose as carbon source and 19mM NH_4_Cl under aerobic and anaerobic conditions (gas atmosphere 7% H_2_, 10% CO_2_, 83% N_2_). Samples were washed twice using ddH_2_O to clear off all carbon and nitrogen residues in the media. Bacterial suspensions were prepared in minimal media M9 without carbon source and NH_4_Cl as nitrogen source for PM1 and PM2A and in minimal media M9 without nitrogen source and pyruvate as carbon source for PM3B. Cell densities were adjusted to OD600nm = 0.02 and plates were inoculated with 100 µl bacterial suspension for 24 hours at 37°C under constant orbital shaking. Tetrazolium dye absorption (OD590nm) was measured every 10 minutes. The mean absorption and corresponding standard deviation was calculated for every substrate well from three independent experiments. Strain specific foldchanges in the negative control well (A1, media control without carbon or nitrogen source) were subtracted as background from each substrate well.

On basis of the Biolog MicroArrays PM1 and PM2A, customized 96-well microplate assays with 41 carbon sources were designed. Minimal media without carbon source was supplemented with each of the carbon sources 0.2% w/v and 230 µl per volume were distributed in duplicates in a flat bottom 96-well plate and stored until usage covered with foil at -20°C. Minimal media without carbon source was used as a blank in the remaining wells. Before usage the plates were incubated without foil but with lid at 37°C until completely thaw. Plates were inoculated with 20 µl bacterial suspension and growth was detected at OD600nm for 24h, as described before.

### Curation of a metabolic network to capture SL1344 observed physiology

Experimental data and evidence were used to curate a workable model to capture the SL1344 metabolism. Two different sources of information were used; growth assays and metabolomics data. Growth assay data on single carbon and nitrogen sources were used to assess and enhance the quality of the network. On top of that, the extracellular space of the reconstruction was expanded to account for fourteen more metabolites that are relevant to the murine gut environment. In absence data, the biomass reaction as well as the growth and non-growth associated ATP requirements used in this study were directly adopted from the STM_v1_0 GEM.

To use the growth assay data, the different compounds were mapped to the metabolite identifiers in the model and the KEGG database. In the absence of other information, the import of the compounds was modeled with a proton symport mechanism to examine whether the model can or cannot predict growth on them while accounting for a minimum amount of energy for the uptake (i.e., import of one proton is equal to a quarter of the requirement to produce 1 mole of ATP, assuming an ATP synthase operating with 4 protons) (S7 Data). For the dipeptides that were not part of the model, we simulated their uptake through a proton symport and their hydrolysis to the respective amino acids by a cytosolic dipeptidase. A dipeptidase family member which might serve dipeptide hydrolysis is encoded in the genome of SL1344 (SL1344_0312). Also, reactions were added to the network to model the C4-dicarboxylates succinate, malate, and aspartate transport, through an antiporter [6]. Finally, five reactions were added to the model to capture the catabolism of L-lysine since glucoselysine is a known nitrogen source for SL1344 under aerobic conditions (S4 Table).

The growth curves were used to estimate the growth rate, at different conditions, at the exponential growth phase. These growth rates were used to constrain the model and obtain physiologically relevant upper bounds for the uptake of the compounds. More specifically, for each of the different conditions where growth was observed experimentally, the estimated growth rates were used to constrain the biomass reaction. Subsequently, the uptake of the corresponding compound was minimized, which is equivalent to the optimization of the yield. After this analysis, the maximum allowable uptake rate was set to 25 mmol/gDW-h. Uptake of this order of magnitude has been experimentally [60] observed.

Metabolism was simulated for the different conditions, and the model predictions were compared against the *in vitro* data. For each of the four datasets, we did not consider the cases where low or late growth or high standard deviation among the different samples was observed (S6 Data). The MCC [44] was used as a metric for the accuracy of the model predictions. MCC is a single-value metric that summarizes the confusion matrix (which includes the true positive, false negative, true negative, and false positive model predictions) and allows the evaluation of binary classifications (i.e., growth – no growth in this case). MCC ranges from -1 (total negative correlation) to 1 (total positive correlation), where 0 means that the model behaves as a random guess classifier. MMC values between 0.7 and 1 indicate moderate to significant model performance [61]. Contrary to other metrics (e.g., F1 score and accuracy) MCC produces a high score only if the model performs well in all four confusion matrix categories and accounts for positive and negative elements in the data [62] and can thus handle imbalanced sets. Consequently, MCC is an appropriate metric to evaluate the performance of GEMs that should be able to capture the organism’s ability to catabolize certain metabolites only.

To curate the FP model predictions, reactions were removed from the model. More specifically, we started with a reaction pool of 364 reactions (DB2). Based on the RAVEN workflow, these are reactions of the STM_v1_0 network that are not part of the SL1344 biochemistry, i.e., no catalyzing gene was identified in the genome of SL1344. We performed a reaction essentiality analysis, i.e., each reaction was blocked, one at a time, and the metabolism was simulated for all the different media and the four datasets. We considered a reaction essential if its deletion would decrease the predicted growth rate by at least 90%, compared to the wildtype model. A reaction was removed only if it was essential for the FP cases only and not for any of the TPs.

For the curation of the FN model predictions, the NICEgame workflow [63] was applied. Shortly, the workflow merges the metabolic network of the organism under study with a database, DB3 and KEGG in this case, into a connected network. It will then generate all alternative sets of reactions, of minimum or bigger size, that need to be added to the model from the database to curate a FN model prediction. The addition of a reaction was allowed only if it wouldn’t introduce FPs in the model predictions.

To expand the extracellular space of the reconstruction, the published redGEMX [42] algorithm was used. In the original formulation, the reactions of the model are split into core and non-core reactions. Then the algorithm identifies alternative minimal sets of non-core reactions to connect an extracellular metabolite to any core metabolite. The algorithm was modified to connect the newly added extracellular metabolites to any of the metabolites in the existing model. More specifically, the algorithm minimizes the sum of the database reactions, e.g., DB3, the KEGG database [64], or the ATLASx [65] database, that need to be active for the uptake and/or secretion of a certain metabolite to be active.

Overall, the rules followed to add reactions in the model were: i) Pathways introducing false positives in comparison to Biolog data were discarded, ii) If catalyzing genes were annotated in the SL1344 genome for all the steps of a pathway, all alternative pathways were added (e.g., pathways are identified using DB3). iii) If more than one pathways existed, but only partial information on the catalyzing genes was available (e.g., genes predicted using the BridgIT+ tool), we selected the pathway for which the most reactions had identified catalyzing genes. iv) In cases where no genes were identified, but there was evidence that the compound was linked to SL1344 metabolism, we included pathways based on in-house knowledge of NTS metabolism.

### Identification of putative catalyzing sequences for the newly added biochemistry

We employed the BridgIT^+^ [29] workflow to assign sequences found in the SL1344 genome to newly added reactions, if possible. For each reaction, BridgIT^+^ outputs putative catalyzing sequences of the SL1344 genome registered on the Uniprot [66] database, if any are found. The threshold value to assign a sequence to a reaction is equal to a bit score of 50. BridgIT^+^ predictions with a lower bit score are referred to as *low similarity.*

### Identification of the nutritional requirements for NTS growth in the murine gut

For the generation of the iMMs, we employed the published [42] formulation. The iMM algorithm is based on a MILP formulation that identifies the minimum set of extracellular metabolites (i.e., minimal set of active boundary reactions) necessary to support a metabolic task, in this case growth. We performed an iMM analysis for the experimentally observed growth rate of SL1344 in LCM mice that is equal to 0.12 h^-1^. Minerals are included by default to all the iMMs. To evaluate the iMMs with respect to the biomass yield, we constrained the upper bound of total carbon and total nitrogen uptake. We used 60 mmol carbon/gDW-h, equivalent to a flux of 10 mmol glucose/gDW-h, and 2 mmol nitrogen/gDW-h, equivalent to a flux of 2 mmol ammonia/gDW-h based on the optimal ratio of glucose to ammonia as calculated using the model. The carbon to nitrogen uptake flux ratio was approximated based on the optimal glucose to ammonia utilization ratio. The iMMs were then categorized in low and high yield based on the optimal growth rate, under the same total carbon and nitrogen uptake constraint; media compositions with optimal growth rate greater or equal to 0.054 h^-1^ (the 0.90 quantile was used as an arbitrary threshold to define high yield supporting media) are considered as high yield whereas media compositions the rest are considered as low yield. For the generation of the essential metabolic byproduct profiles, we used we generated the minimal sets of active secretions at optimal biomass yield for each minimal media composition.

Since the biomass composition, growth and non-growth associated maintenance energy (GAM and NGAM, respectively) were adapted from the STM_v1_0 GEM and were not tailored to the SL1344 strain, a sensitivity analysis was performed. In this analysis, we generated 100 alternative sets of biomass composition, GAM and NGAM, allowing values to diverge up to 20% of the initial value (S10 Data). For each of these 100 sets, we generated the iMMs of minimal and the subsequent size (S10 Data). In these perturbed models, the metabolites appearing in the iMMs of minimal and subsequent size represent at least 76% of the metabolites captured in the iMMs of the same sizes with the iNTS_SL1344 GEM, and in 22 of the models the iMM metabolite coverage is the same as in the iNTS_SL1344 GEM (S10 Data).

### *In vitro* validation of the iMM predictions

The curated and context-specific metabolic network to identify metabolites essential for the growth of SL1344 in the murine gut. An *in silico* Minimal Media (iMM) analysis was performed for anaerobic conditions to suggest putative nutrients for SL1344 within a list of 81 metabolites relevant to NTS physiology in the murine gut environment. Starting from the rich environment, including inorganics, amino acids, carboxylic acids, sugars, and bile acids, all alternative iMMs of minimal size were generated, i.e., sets of the minimal number of compounds required to achieve certain growth. The iMM predictions were pruned to media compositions with the same inorganic metabolites using the M9 recipe but different carbon and nitrogen sources. The number of tested iMMs was limited by the capacity of the wet lab, the availability of the compounds and how easy it is to work with them. The concentration of the assumed carbon source was constrained to be equimolar to 0.4 g of glucose/ 100 mL of media and the total nitrogen equimolar to 0.1 g of NaCl/ 100 mL of media. Six different *high yield* combinations of carbon and nitrogen sources were used in M9 media as described for the Biolog growth assays. The *low yield* media set consisted of nine different combinations of carbon and nitrogen sources in M9 media (S11 Table). 230 µl per well of each media were distributed in triplicates for blanks and in triplicates for growth detection in a flat bottom 96-well plate. Bacterial suspensions were prepared and OD600nm was measured as described before under anaerobic conditions for 24h.

### *In silico* gene knockout studies

Single gene essentiality analyses with thermodynamic constraints were performed for each of the predicted minimal media (iMMs) and the minimal media used for the single carbon and nitrogen source growth assays and anaerobic conditions. For the latter, we considered only the media compositions that the model correctly predicts growth, i.e., the TP cases. A gene was considered essential *in silico* if the growth rate of the knockout mutant was less than 10% of the growth rate of the wildtype. We report the relative fitness of the KO strain compared to the WT strain, which means the optimal growth rate of each KO strain in the media conditions under study to the growth rate of the WT strain on the same medium.

### Software

This work was supported by EPFL through the use of the facilities of its Scientific IT and Application Support Center. We performed the genome annotation using the RAVEN toolbox in MATLAB 2019b. The annotation was done on a high-performance computing cluster of 408 nodes. The analysis was performed on Mac Pro 32 GB in MATLAB 2017a and IBM ILOG Cplex 12.7.1 as a solver.

## Supporting information

S1 Data The DB1 database contains the functional genome annotation of SL1344 using the published GEM STM_v1_0 of the strain LT2 and contains the common metabolic genes and associated reactions of the two strains. (XLSX)

S2 Data The DB2 contains the common metabolic genes and associated reactions of STM_v1_0 GEM that are unique to the strain LT2. (XLSX)

S3 Data The DB3 database contains the functional genome annotation of SL1344 using the published KEGG database. (XLSX)

S4 Data Comparison of reaction content of the more and less conserved main metabolic subsystems for the STM_v1_0 GEM and DB1. The subsystems definition is based on the STM_v1_0 GEM.
(XLSX)

S5 Data Common genes, reactions and metabolites for DB1, DB3 and STM_v1_0. (XLSX)

S6 Data Comparison of the STM_v1_0 and iNTS_SL1344 GEMs predictions against the Biolog data. (XLSX)

S7 Data Transport mechanisms used in the iNTS_SL1344 GEM. (XLSX)

S8 Data Composition of the predicted minimal media (iMMs) and optimal growth rate achieved on each iMM. Sets of the minimal byproducts for each iMM.
(XLSX)

S9 Data Data of gene essentiality for different minimal media compositions. (XLSX)

S10 Data The file contains data from the sensitivity analysis on biomass composition, GAM, and NGAM with respect to the iMM model analysis results. It includes the perturbed parameter values, the resulting iMM models, and the list of metabolites that appear in iMM models of the same sizes in the iNTS_SL1344 network but are absent in the perturbed models.
(XLSX)

S1 Text Information on simulations to investigate why some compounds can support growth under aerobic conditions but not in the absence of oxygen. (DOCX)

S2 Text Generation of reaction databases used to curate the iNTS_SL1344 GEM. (DOCX)

S1 Fig Compounds that can serve as single carbon sources for SL1344 under aerobic and anaerobic conditions, based on the Biolog Phenotype MicroArrays PM1 and PM2A. The nitrogen source is ammonium.
(TIF)

S2 Fig Compounds that can serve as single nitrogen sources for SL1344 under aerobic and anaerobic conditions, based on the Biolog Phenotype MicroArray PM3B. The carbon source is pyruvate.
(TIF)

S3 Fig Compounds that can serve as single carbon sources for SL1344 under aerobic and anaerobic conditions, based on in-house made microarrays. The nitrogen source is ammonium.
(TIF)

S4 Fig Comparison of Biolog and in-house made phenotype microarrays for compounds that can serve as single carbon sources for SL1344 under aeorobic and anaerobic conditions. The nitrogen source is ammonium.
(TIF)

S5 Fig Representative flux distribution for aerobic growth on (A) glucose, (B) pyruvate, (C) succinate, (D) L-lactate, (E) glycerol, and (F) L-glutamate for optimal growth with maximum allowable carbon source uptake of 25 mmol/gDW-h. The flux distributions plotted here are the average of 200,000 samples obtained using an artificially centered hit and run (ACHR) sampling algorithm. The networks are plotted using the Escher map web application (https://doi.org/10.1371/journal.pcbi.1004321). Succinate enters metabolism through the TCA cycle and is converted to fumarate by succinate dehydrogenase (SUCDi), linking TCA to oxidative phosphorylation (C). When this happens, 2 protons and 2 electrons are removed which are taken by ubiquinone-8 and reduce it to ubiquinol-8. When oxygen is available, it acts as a final electron acceptor and ubiquinol-8 is re-oxidized to ubiquinone (CYTBO3_4pp and CYTBDpp), maintaining balance. Similarly, L-lactate is transported into the cell and is converted to pyruvate through lactate dehydrogenase (L_LACD2 and L_LACD3) that is coupled with the quinone pool (D). In both cases, in the absence of oxygen or another electron acceptor, the quinols cannot be re-oxidized to quinones, resulting in no growth phenotypes when succinate or L-lactate serve as single carbon sources. The utilization of glycerol (E) leads to additional NADH and NADPH production through the glycerol dehydrogenase and the glycerol-3-phosphate dehydrogenase (GLYCDx and G3PD2) compared to glucose utilization (A). In aerobic conditions, oxygen acts as the electron acceptor and enables the re-oxidation of quinols to quinones (CYTBO3_4pp, CYTBDpp and CYTBD2pp). Then NADH and NADPH can be oxidized to NAD^+^ and NADP^+^, respectively, transferring 2 electrons to the quinones (NADH16pp, NADH5, NADPHQR2, NADH17pp, NADH10, NADH9 and NADPHQR4). In the absence of oxygen, the generated NADH and NADPH cannot be oxidized leading to redox imbalance and no growth. L-glutamate is converted to oxoglutarate (GLUDy and ASPTA) and enters the TCA cycle to produce malate that is converted to pyruvate (ME1 and ME2), that fuels gluconeogenesis (F). The steps to convert L-glutamate to pyruvate generate excessive NADH and NADPH to pyruvate utilization (B), which cannot be oxidized in the absence of oxygen, leading to no growth phenotype.
(TIF)

S6 Fig (A) Gene and reaction overlap of the STM_v1_0 GEM and the reaction databases DB1 and DB3. Performing a functional genome annotation using the KEGG database identifies metabolic capabilities that are unique to the SL1344 strain compared to the LT2 strain. 1,117 metabolic genes (*) are orthologous to 1,104 genes included in the STM_v1_0 GEM whereas no orthologous gene was identified for 166 genes in the STM_v1_0 GEM. (B) Common reactions between STM_v1_0 and the DB1. The main metabolic subsystems (as defined in the STM_v1_0 GEM) are highly preserved between the LT2 and SL1344 strains.
(TIF)

S7 Fig Statistics for the minimal byproducts for each one of the predicted iMMs at optimal biomass yield. The compounds in green appear only with low-yield minimal media.
(TIF)

S8 Fig Frequency of substrate-byproduct pairs appearance in all the predicted minimal media (iMMs) for five selected byproducts. Succinate, acetate, formate and ethanol appear in the minimal byproducts for the majority of media compositions, whereas lactate appears in only one alternative where methyl-D-galactoside, glutamine and glycine are the carbon and nitrogen sources. Substrate-byproduct associations can help us identify metabolic signatures of Salmonella. The full dataset is provided in the S8 Data.
(TIF)

S1 Table Modeled ATP yield under aerobic and anaerobic conditions for compounds that can serve only as carbon sources under aerobic conditions and comparison with glucose and pyruvate. (XLSX)

S2 Table Compounds relevant to STM metabolism in the murine environment and their identifiers in the model. (XLSX)

S3 Table Compounds of interest and the minimal pathways to connect them to the metabolism of SL1344. (XLSX)

S4 Table Added and removed reactions to reconcile the model predictions with the growth assays data. (XLSX)

S5 Table Gene predictions for added reactions. (XLSX)

S6 Table Gene predictions for orphan reactions in the STM network. (XLSX)

S7 Table Examples of essential orphan reaction under glucose minimal media and aerobic conditions. (XLSX)

S8 Table Catalyzing genes for the reactions added to the model from DB3 (i.e., genome annotation for SL1344 using the RAVEN workflow and the KEGG database). (XLSX)

S9 Table Competitive index values for SL1344 mutants in the murine gut. The data are retrieved from doi: 10.1016/j.chom.2020.04.013.
(XLSX)

S10 Table Thermodynamic properties integrated into the iNTS_SL1344 network. (XLSX)

S11 Table Composition of experimentally tested minimal media (iMM). (XLSX)
