# Supplementary material for: Metabolic network reconstruction as a resource for analyzing Salmonella Typhimurium SL1344 growth in the mouse intestine
Source: PLoS Comput Biol. 2025 Mar 11;21(3):e1012869. doi: 10.1371/journal.pcbi.1012869 (PMC11925469; doi:10.1371/journal.pcbi.1012869)
Supplement: S1 Text — (DOCX) [file pcbi.1012869.s011.docx]

***Supplementary Note 1***

A series of simulations were performed to investigate why some compounds can support growth under aerobic conditions but not in the absence of oxygen. We examined the effect of maximum uptake rate and ATP maintenance requirements. Details on these simulations are provided below.

The initial allowable nutrient uptake rate was 25 mmol / gDW-h for all media compounds, and the lower bound for ATP maintenance was set at 8.39 mmol / gDW-h, for both aerobic and anaerobic conditions. For the discussed compounds (D-alanine, L-asparagine, and L-alanylglycine) under aerobic conditions this ATP production can be achieved since there is growth. Under anaerobic conditions, we let the uptake rate of the carbon source unbounded (allowed uptake rate 1000 mmol / gDW- h), we set a lower bound to the biomass reaction (0.001 h^-1^) and minimized the uptake rate of the carbon source. Growth could only be achieved when D-alanine was the carbon source (minimum required uptake to achieve μ = 0.001 h^-1^ was found to be 31.6065 mmol/gDW-h). For these simulations, the ATP maintenance lower bound was kept at 8.39 mmol / gDW-h. We also performed another set of similar simulations to examine the effect of the lower bound for ATP maintenance (the maximum uptake rate was kept at 25 mmol/gDW-h) under anaerobic conditions. This time, μ = 0.001 h^-1^ could be achieved on L-asparagine when maximum lower bound for ATP maintenance was 6.1672 mmol/gDW-h, on D-alanine when maximum lower bound for ATP maintenance was 7.3995 mmol/gDW-h and on L-alanylglycine when maximum lower bound for ATP maintenance was 8.2412 mmol/gDW-h.
