# Supplementary material for: Metabolic network reconstruction as a resource for analyzing Salmonella Typhimurium SL1344 growth in the mouse intestine
Source: PLoS Comput Biol. 2025 Mar 11;21(3):e1012869. doi: 10.1371/journal.pcbi.1012869 (PMC11925469; doi:10.1371/journal.pcbi.1012869)
Supplement: S2 Text — (DOCX) [file pcbi.1012869.s012.docx]

***Supplementary Note 2***

To generate DB1 and DB2, the translated coding sequences for LT2 and SL1344 were obtained from the NCBI database[1]. To compare the two genomes, a bidirectional BLASTP analysis was performed using the RAVEN[2,3] toolbox. A cutoff value, i.e., E-value, equal to 10^-30^ was used to evaluate the similarity of the different sequences. Using the results of this sequence comparison, we divided the genes and reactions of the STM_v1_0 GEM into two categories; the genes and reactions in the first category are common for the two strains (DB1) whereas the genes and reactions in the second category are only associated to the metabolism of the LT2 strain (DB2). DB1 contains 1,117 metabolic genes and 1,803 reactions among 1,065 unique metabolites (S6A Fig and S5 Data). These 1,117 metabolic genes are orthologous to 1,104 genes included in the STM_v1_0 GEM (S5 Data), whereas no orthologous gene was identified for 166 genes in the STM_v1_0 GEM (S6 Fig). Furthermore, there are 201 biotransformation reactions that are not included in the DB1, since no homologue of the encoding gene (or at least one of the encoding genes in the case more than one genes are necessary for the reaction to occur in the STM_v1_0 GEM, e.g., gene1 AND gene2 gene-reaction rule assigned to the reaction) was identified in the SL1344 genome. These 201 reactions, along with the orphan reactions, i.e., reactions that are not associated with any gene, in the LT2 network, 364 reactions in total, constitute the DB2 and are candidate reactions to be removed from the model to curate redundant metabolic capabilities. Overall, the two strains (i.e., SL1344 and LT2) appear to differ mainly in nitrogen metabolism and oxidative phosphorylation as defined in the STM_V1_0 GEM with respect to the percentage of the gene-related preserved reactions in each subsystem, whereas the biggest absolute differences concern the transport and cell envelope biosynthesis (S4 Data). Contrary, the alternate carbon metabolism and the anaplerotic pathways as defined in the STM_V1_0 GEM are highly preserved (S6B Fig and S4 Data).

Although using the GEM of LT2 to build a genome-scale model of the closely related SL1344 organism has the advantage of including in the reconstruction information that has been obtained through manual curation, it has an obvious drawback. The novel reconstruction can only contain information that is included in the template model. To surpass this, we have performed functional annotation of the genome SL1344 and the KEGG[4–6] version 2018 as a database using an E-value of 10^-10^ as a threshold. This process allows us to generate DB3 that can be used to extract this missing information. DB3 contains 1,468 reactions among 1,526 metabolites associated with 1,145 genes (S6A Fig a S3 Data and S5 Data).

***References***

1. Schoch CL, Ciufo S, Domrachev M, Hotton CL, Kannan S, Khovanskaya R, et al. NCBI Taxonomy: a comprehensive update on curation, resources and tools. Database. 2020 Jan 1;2020.

2. Wang H, Marcišauskas S, Sánchez BJ, Domenzain I, Hermansson D, Agren R, et al. RAVEN 2.0: A versatile toolbox for metabolic network reconstruction and a case study on Streptomyces coelicolor. Ouzounis CA, editor. PLoS Comput Biol. 2018 Oct 18;14(10):e1006541.

3. Agren R, Liu L, Shoaie S, Vongsangnak W, Nookaew I, Nielsen J. The RAVEN Toolbox and Its Use for Generating a Genome-scale Metabolic Model for Penicillium chrysogenum. Maranas CD, editor. PLoS Comput Biol. 2013 Mar 21;9(3):e1002980.

4. Kanehisa M, Goto S. KEGG: Kyoto Encyclopedia of Genes and Genomes. Vol. 28, Nucleic Acids Research. Oxford University Press; 2000. p. 27–30.

5. Kanehisa M, Furumichi M, Sato Y, Ishiguro-Watanabe M, Tanabe M. KEGG: integrating viruses and cellular organisms. Nucleic Acids Res. 2021 Jan 8;49(D1):D545–51.

6. Kanehisa M. Toward understanding the origin and evolution of cellular organisms. Protein Science. 2019 Nov 1;28(11):1947–51.
