# Supplementary figures and images for: Metabolic network reconstruction as a resource for analyzing Salmonella Typhimurium SL1344 growth in the mouse intestine
Source: PLoS Comput Biol. 2025 Mar 11;21(3):e1012869. doi: 10.1371/journal.pcbi.1012869 (PMC11925469; doi:10.1371/journal.pcbi.1012869)

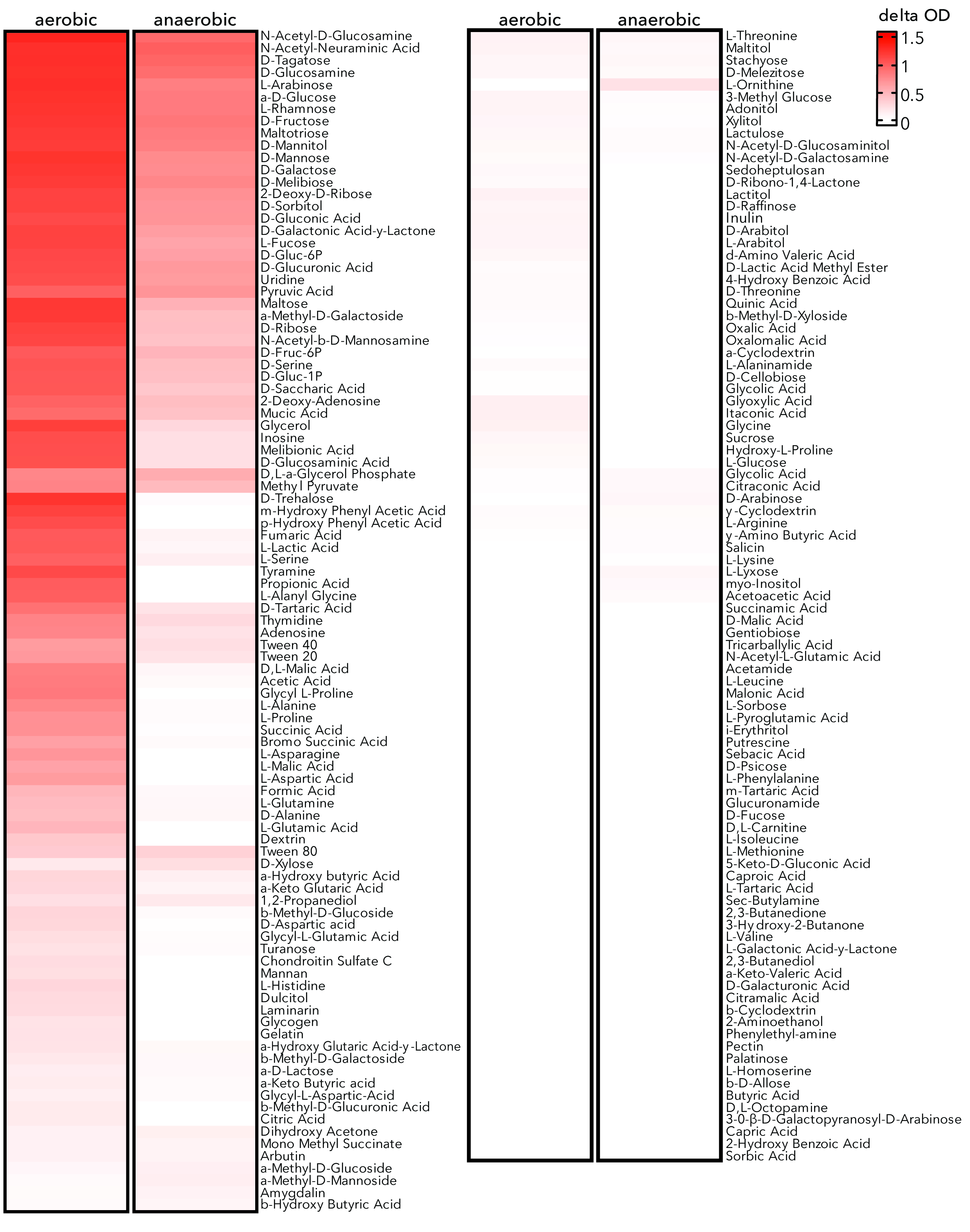

Supplement: S1 Fig — The nitrogen source is ammonium. (TIF) [file pcbi.1012869.s013.tif]

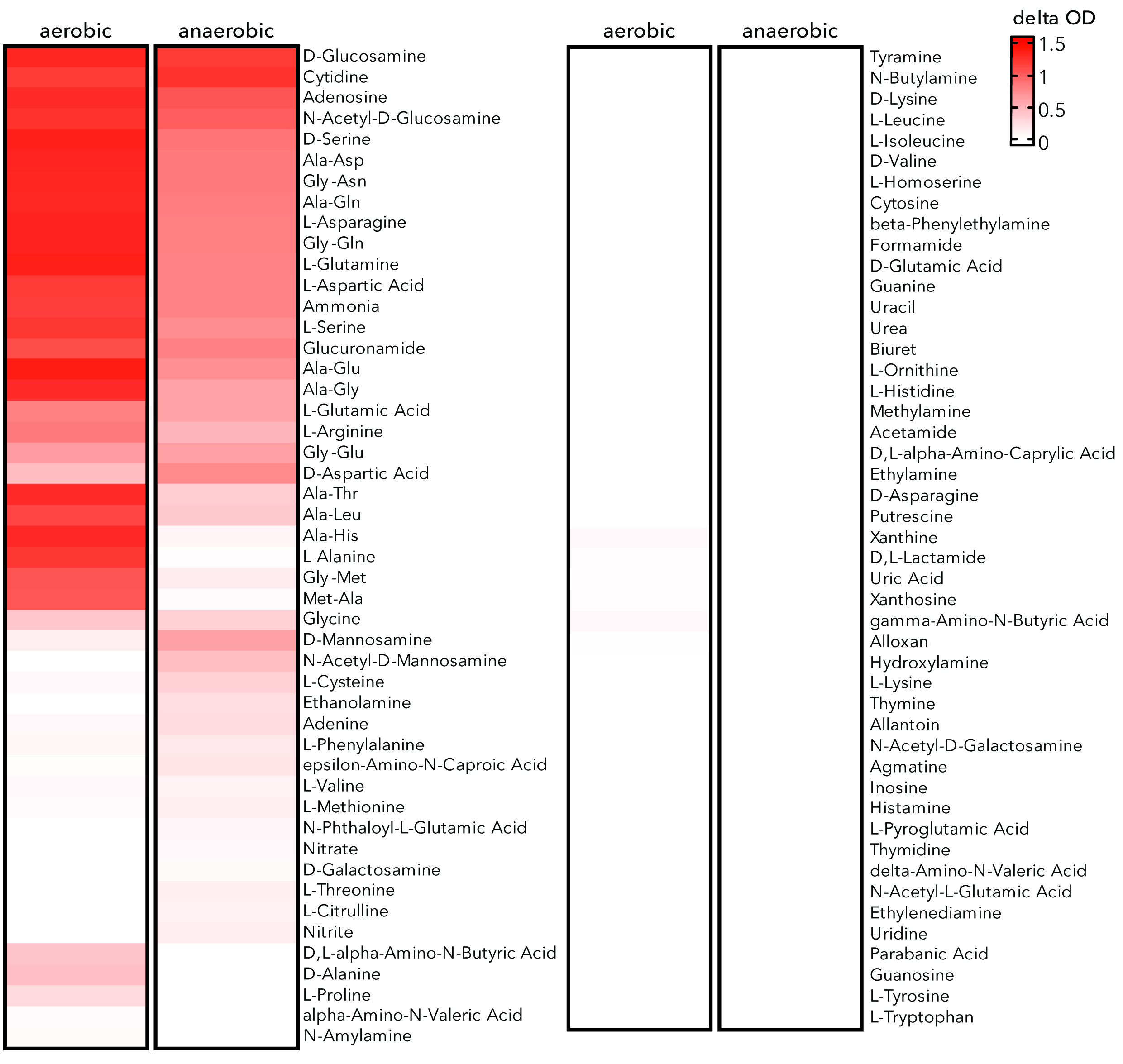

Supplement: S2 Fig — The carbon source is pyruvate. (TIF) [file pcbi.1012869.s014.tif]

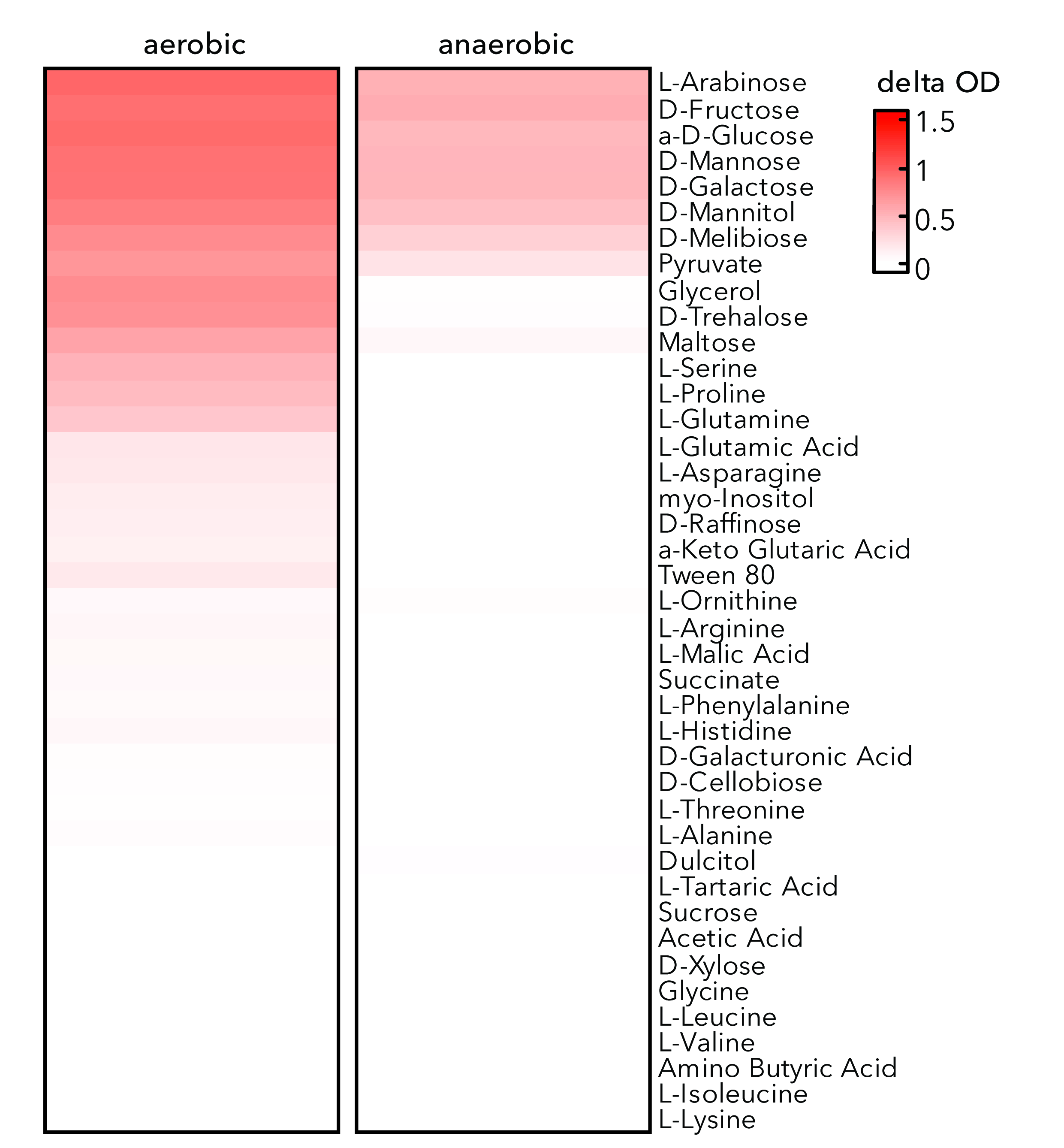

Supplement: S3 Fig — The nitrogen source is ammonium. (TIF) [file pcbi.1012869.s015.tif]

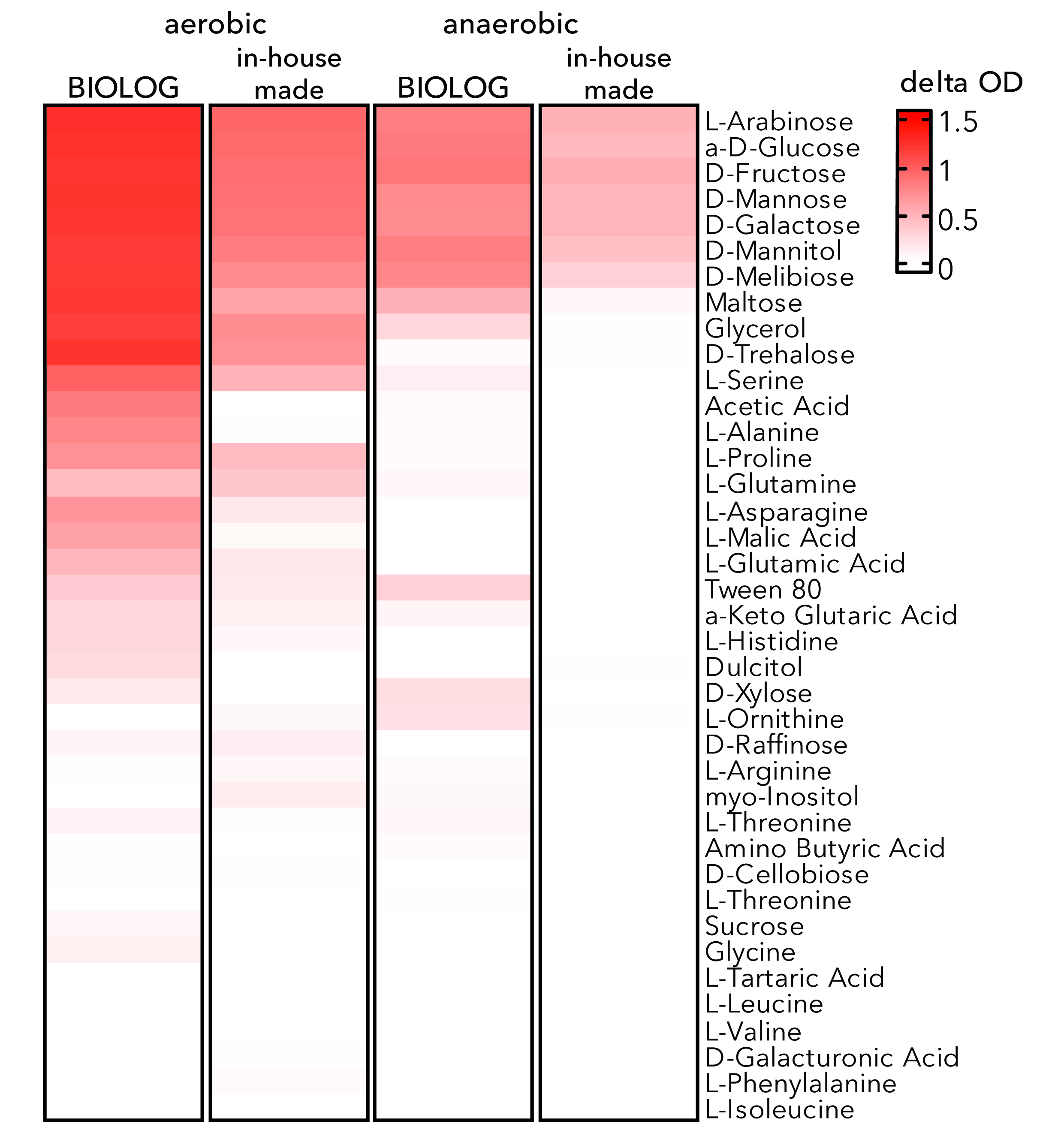

Supplement: S4 Fig — The nitrogen source is ammonium. (TIF) [file pcbi.1012869.s016.tif]

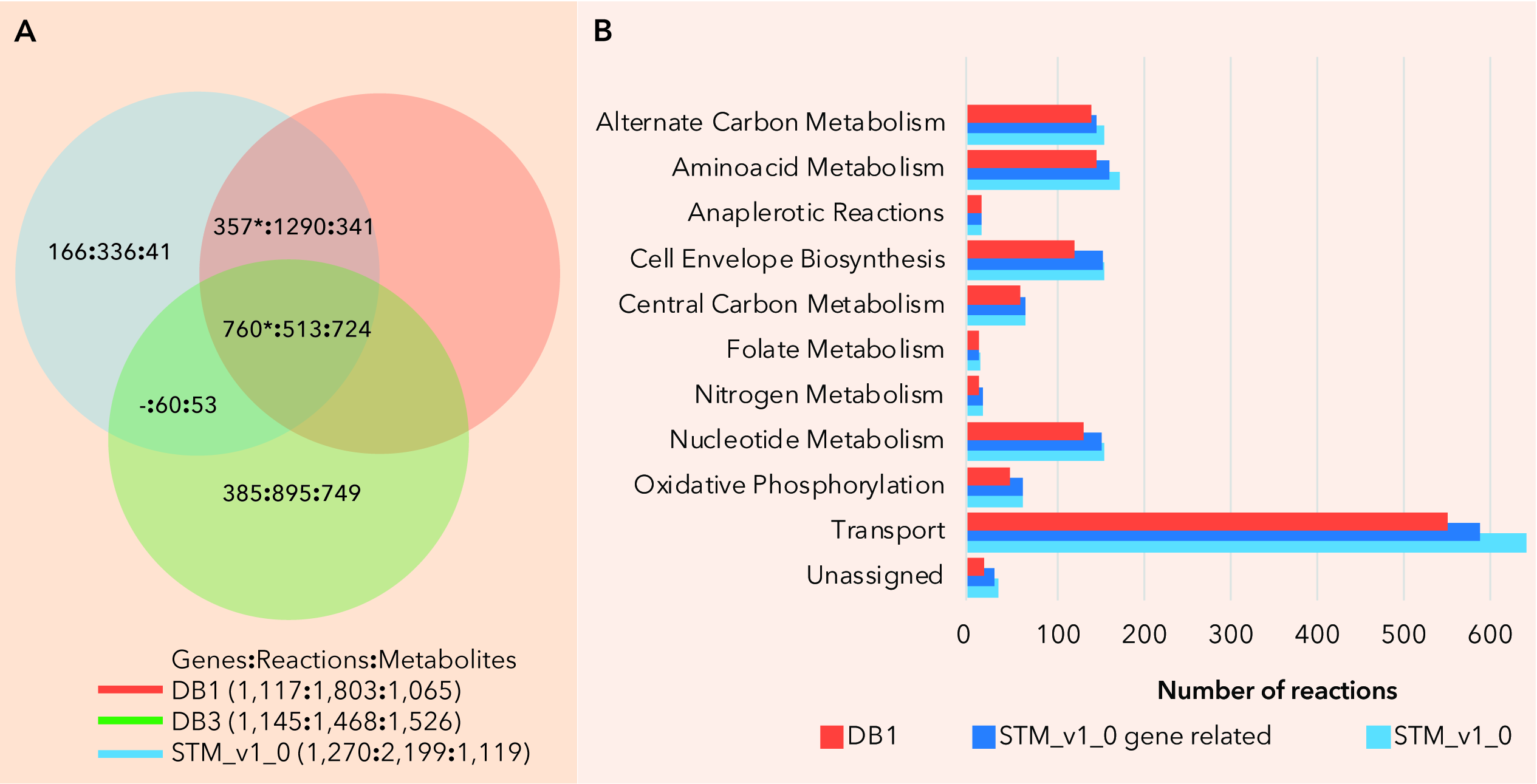

Supplement: S6 Fig — Performing a functional genome annotation using the KEGG database identifies metabolic capabilities that are unique to the SL1344 strain compared to the LT2 strain. 1,117 metabolic genes (*) are orthologous to 1,104 genes included in the STM_v1_0 GEM whereas no orthologous gene was identified for 166 genes in the STM_v1_0 GEM. (B) Common reactions between STM_v1_0 and the DB1. The main metabolic subsystems (as defined in the STM_v1_0 GEM) are highly preserved between the LT2 and SL1344 strains. (TIF) [file pcbi.1012869.s018.tif]

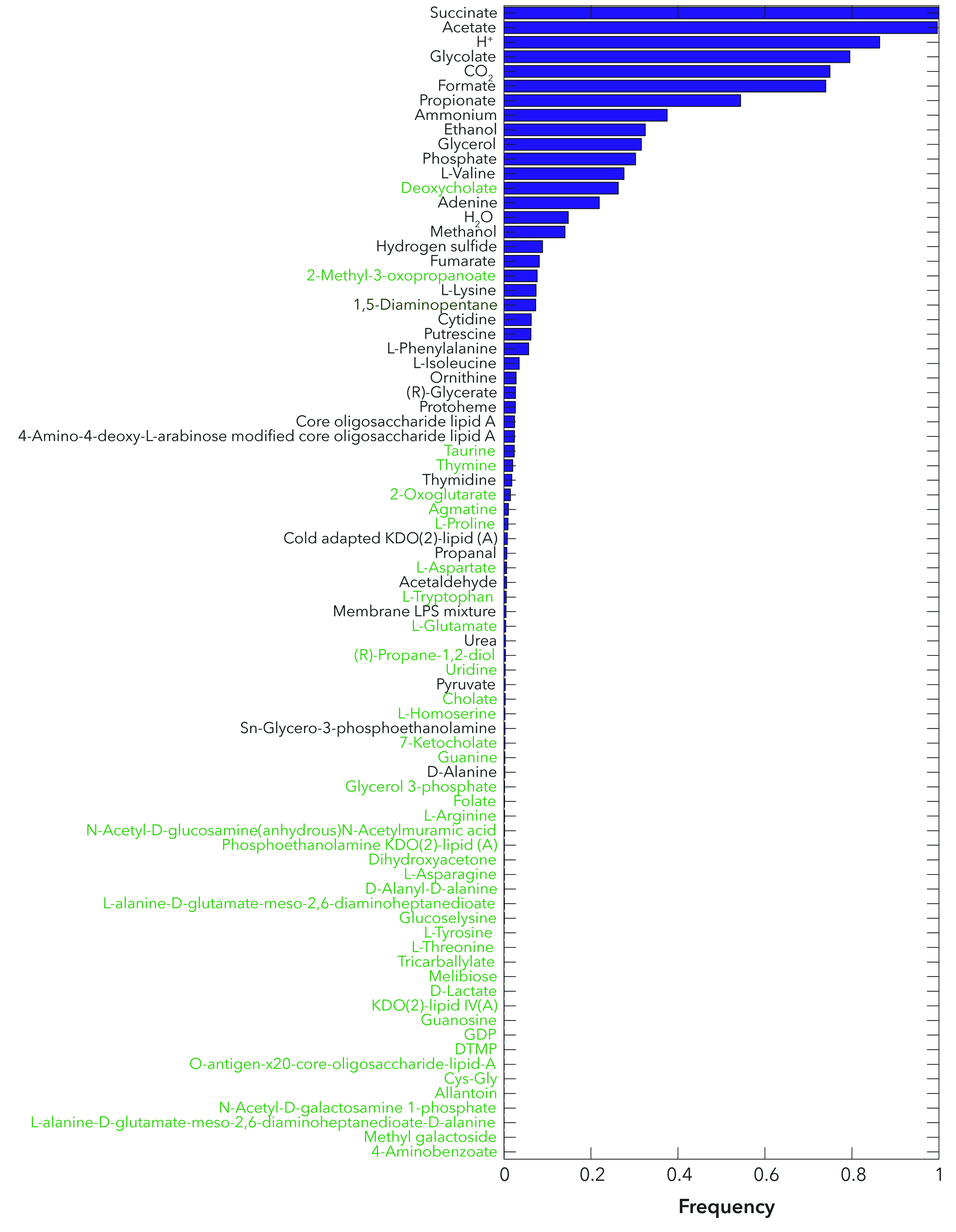

Supplement: S7 Fig — The compounds in green appear only with low-yield minimal media. (TIF) [file pcbi.1012869.s019.tif]

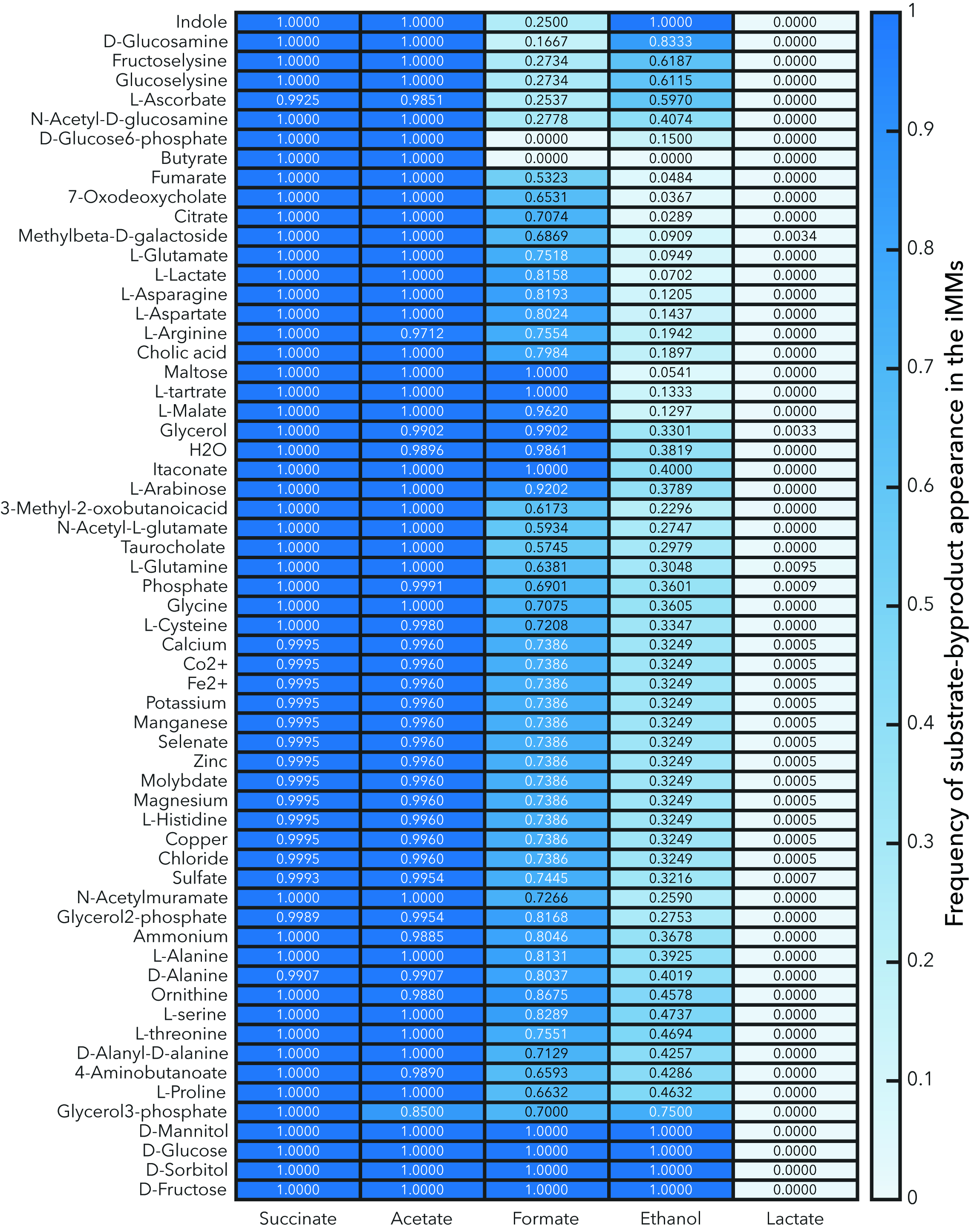

Supplement: S8 Fig — Succinate, acetate, formate and ethanol appear in the minimal byproducts for the majority of media compositions, whereas lactate appears in only one alternative where methyl-D-galactoside, glutamine and glycine are the carbon and nitrogen sources. Substrate-byproduct associations can help us identify metabolic signatures of Salmonella. The full dataset is provided in the S8 Data. (TIF) [file pcbi.1012869.s020.tif]
